# The roles of *SMYD4* in epigenetic regulation of cardiac development in zebrafish

**DOI:** 10.1371/journal.pgen.1007578

**Published:** 2018-08-15

**Authors:** Deyong Xiao, Huijun Wang, Lili Hao, Xiao Guo, Xiaojing Ma, Yanyan Qian, Hongbo Chen, Jing Ma, Jin Zhang, Wei Sheng, Weinian Shou, Guoying Huang, Duan Ma

**Affiliations:** 1 Key Laboratory of Metabolism and Molecular Medicine, Ministry of Education, Department of Biochemistry and Molecular Biology, Collaborative Innovation Center of Genetics and Development, Institutes of Biomedical Sciences, School of Basic Medical Sciences, Fudan University, Shanghai, China; 2 Shanghai Key Lab of Birth Defect, Children’s Hospital of Fudan University, Shanghai, China; 3 Pediatric Heart Center, Children’s Hospital of Fudan University, Shanghai, China; 4 Cardiovascular Developmental Biology Group, Herman B Wells Center for Pediatric Research, Indiana University School of Medicine, Indianapolis, IN, United States of America; Mayo Clinic, UNITED STATES

## Abstract

SMYD4 belongs to a family of lysine methyltransferases. We analyzed the role of *smyd4* in zebrafish development by generating a *smyd4* mutant zebrafish line (*smyd4*^L544Efs*1^) using the CRISPR/Cas9 technology. The maternal and zygotic *smyd4*^L544Efs*1^ mutants demonstrated severe cardiac malformations, including defects in left-right patterning and looping and hypoplastic ventricles, suggesting that *smyd4* was critical for heart development. Importantly, we identified two rare *SMYD4* genetic variants in a 208-patient cohort with congenital heart defects. Both biochemical and functional analyses indicated that *SMYD4(G345D)* was pathogenic. Our data suggested that smyd4 functions as a histone methyltransferase and, by interacting with HDAC1, also serves as a potential modulator for histone acetylation. Transcriptome and bioinformatics analyses of *smyd4*^L544Efs*1^ and wild-type developing hearts suggested that *smyd4* is a key epigenetic regulator involved in regulating endoplasmic reticulum-mediated protein processing and several important metabolic pathways in developing zebrafish hearts.

## Author summary

SMYD4 belongs to a SET and MYND domain-containing lysine methyltransferase. In zebrafish, *smyd4* is ubiquitously expressed in early embryos and becomes enriched in the developing heart at 48 hours post-fertilization (hpf). We generated a *smyd4* mutant zebrafish line (*smyd4*^L544Efs*1^) using the CRISPR/Cas9 technology. The maternal and zygotic *smyd4*^L544Efs*1^ mutants demonstrated a strong defect in cardiomyocyte proliferation, which led to a severe cardiac malformation, including left-right looping defects and hypoplastic ventricles. More importantly, two rare genetic variants of *SMYD4* were enriched in a 208-patient cohort with congenital heart defects. Both biochemical and functional analyses indicated that *SMYD4(G345D)* was highly pathogenic. Using mass spectrometric analysis, SMYD4 was shown to specifically interact with histone deacetylase 1 (HDAC1) via its MYND domain. Altered di- and tri-methylation of histone 3 lysine 4 (H3K4me2 and H3K4me3) and acetylation of histone 3 in *smyd4*^L544Efs*1^ mutants suggested that *smyd4* plays an important role in epigenetic regulation. Transcriptome and pathway analyses demonstrated that the expression levels of 3,856 genes were significantly altered, which included cardiac contractile genes, key signaling pathways in cardiac development, the endoplasmic reticulum-mediated protein processing pathway, and several important metabolic pathways. Taken together, our data suggests that *smyd4* is a key epigenetic regulator of cardiac development.

## Introduction

Protein post-translational modifications (PTMs) are critical for the biological function of proteins. Histone modification is a common epigenetic mechanism that plays essential roles in the regulation of chromatin structure and gene expression. Different types of histone modifications, which are mediated by a series of specific enzymes, can either enhance or inhibit transcription to regulate specific cellular functions or signaling pathways. SET and MYND domain-containing proteins (SMYDs) belong to a unique family of histone lysine methyltransferases. This family is composed of five members, including SMYD1, SMYD2, SMYD3, SMDY4, and SMYD5. These proteins share a Su(var)3-9, an Enhancer-of-zeste and Trithorax (SET) domain with lysine-specific methyltransferase activity, a Myeloid, Nervy, and DEAF-1 (MYND) domain, and a tetratricopeptide repeat (TPR) domain, which are involved in protein-protein interactions [1–3]. Several biochemical studies and functional analyses showed that SMYDs 1–3 exhibit methyltransferase activities for both histone and non-histone proteins [3–6]. SMYD members are widely present in multiple cell types, including those of skeletal and cardiac muscles [7–9]. Genetic ablation of *Smyd1* in mice led to defects in right ventricular development [8,10]. The knockdown of *smyd1*, *smyd2*, and *smyd3* in zebrafish using morpholino technology also led to cardiac malformation and defects in skeletal and cardiac myofibrillogenesis [11–13]. Zebrafish s*myd5* was recently reported to play important roles in hematopoiesis [14]. Our knowledge of the biological function of SMYD4 remains limited. A previous study found that *dSMYD4* was crucial for muscle development in *Drosophila* [15]. However, the role of *SMYD4* in vertebrate development and its underlying molecular mechanism have not yet been analyzed thoroughly.

Congenital heart diseases (CHDs) are the most common birth defects, with an annual incidence of approximately 1% of newborns worldwide [16]. Heart development involves complex genetic and epigenetic regulation [17–19]. Cardiac-specific ablation of *HDAC1* and *HDAC2* in the murine heart led to aberrant gene expression, contributing to defects in cardiac morphogenesis and contractility [20], suggesting the critical roles of histone modification and epigenetic regulation in cardiac development. The significant enrichment of mutations in genes encoding chromatin modifiers in patients with CHDs provided further evidence to support this notion [21,22].

In this study, we analyzed *smyd4* expression in zebrafish embryos and generated a *smyd4* loss-of-function mutation (*smyd4*^L544Efs*1^) using the CRISPR/Cas9 genome editing technology. We demonstrated that *smyd4* is critical for cardiac malformation in zebrafish development. Two rare missense *SMYD4* variants were identified in patients with CHDs. Both *in vitro* biochemical assays and *in vivo* functional analyses strongly suggested that the variant *SMYD4(G345D)* was pathogenic. Our data suggested that *smyd4* functions as a histone methyltransferase and, by interacting with HDAC1, also serves as a potential modulator for histone acetylation. Transcriptome and bioinformatics analyses of the differentially expressed genes in developing hearts isolated from maternal and zygotic *smyd4*^L544Efs*1^ (MZ*smyd4*^L544Efs*1^) mutant and normal control embryos showed a significant enrichment of the key genes involved in cardiac development and contractile function, and the endoplasmic reticulum-mediated protein processing pathway and several important metabolic pathways. Taken together, our results suggest that *smyd4*, in association with *hdac1*, is an important epigenetic regulator of these critical pathways during zebrafish cardiac development.

## Results

### *smyd4* expression is specifically enriched in the developing heart

**Fig 1:**
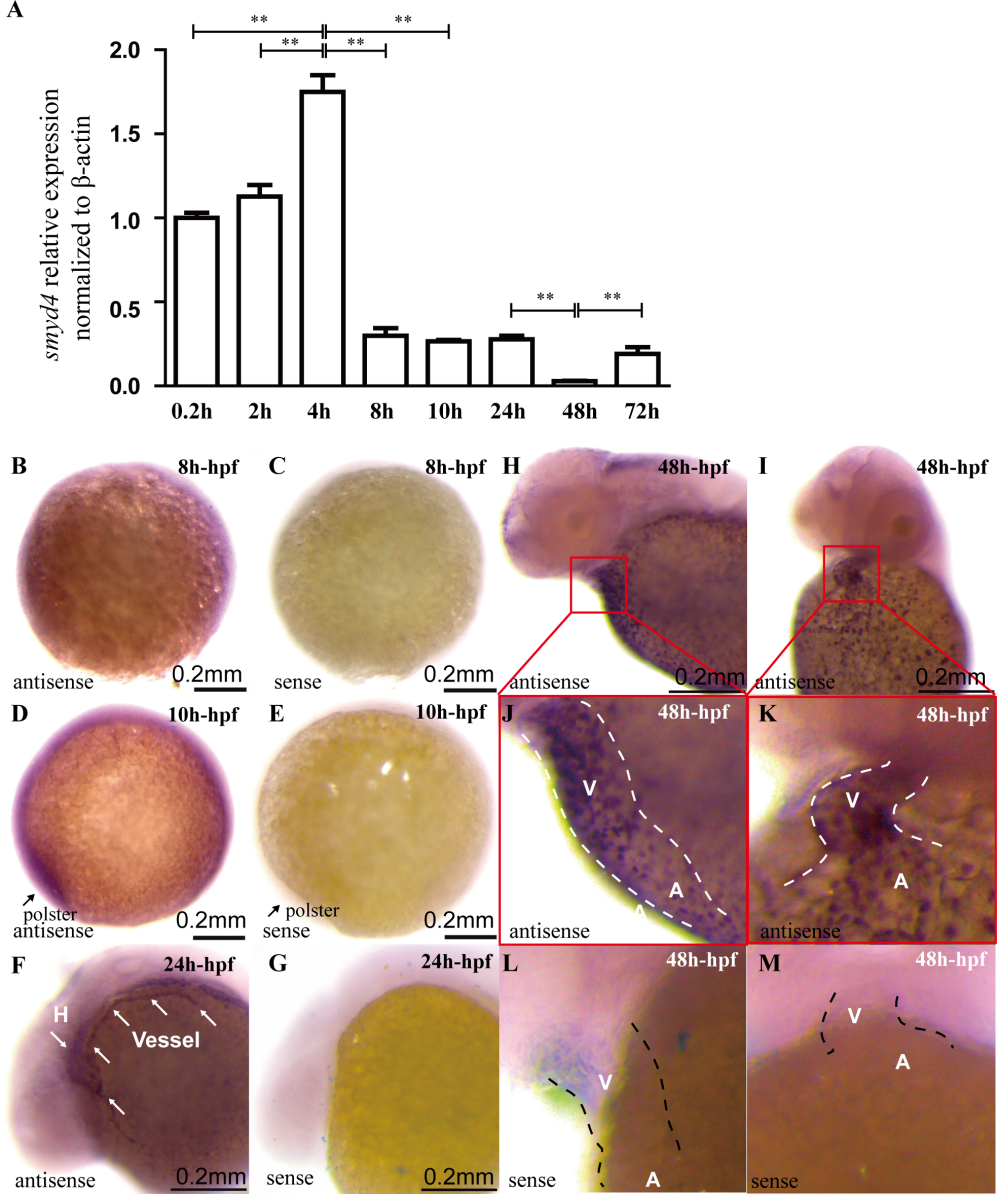
Quantitative RT-PCR and whole-mount *in situ* hybridization and qRT-PCR analyses of *smyd4* expression in zebrafish early embryogenesis. **(A)** The overall expression levels of *smyd4* at 0.2, 2, 4, 8, 10, 24, 48 and 72 hpf were analyzed using qRT-PCR. *smyd4* displayed its highest expression levels during the blastula stage (4 hpf) and was significantly decreased at 48 hpf. At 72 hpf, there was a quick reactivation of *smyd4* expression. **(B-C)** During the mid-gastrula stage (8 hpf), *smyd4* was ubiquitously expressed in the mid-gastrula embryo (8 hpf). **(D, E)** *smyd4* was expressed ubiquitously at 10 hpf, with the highest expression levels found at the polster. **(F, G)** *smyd4* was abundant in the heart and blood vessel at 24 hpf. **(H-M)** Lateral and frontal view of *smyd4* expression at 48 hpf. *smyd4* became more enriched in the developing hearts at 48 hpf, and the ventricle had a higher expression level than that in the atrium. A *smyd4* sense probe was used as the negative control for the *in situ* hybridization analysis.

**Fig 2:**
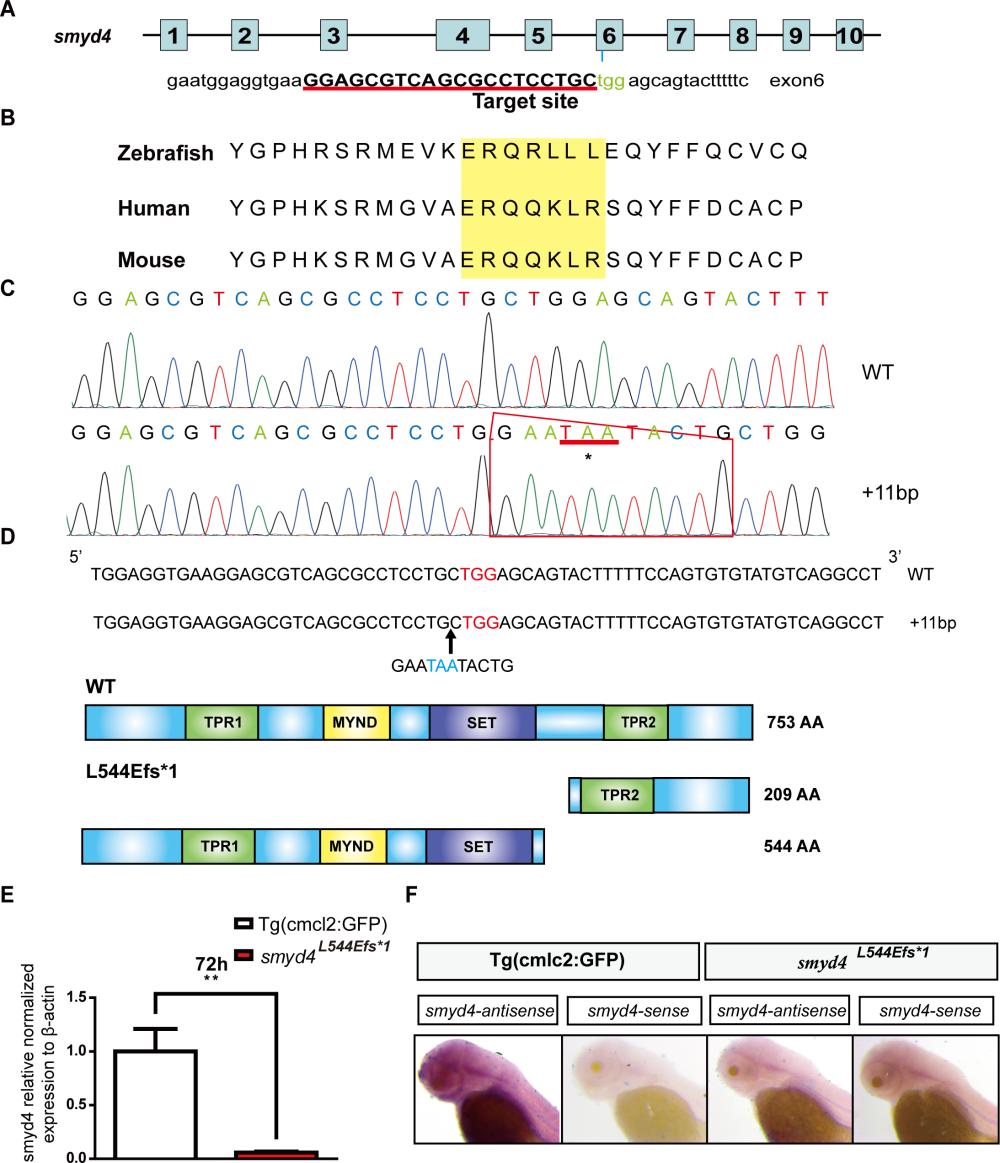
The generation of *smyd4*–deficient zebrafish using the CRISPR/Cas9 technology. **(A)** Schematic diagram of the *smyd4* gene structure and the sgRNA3 sequence for the exon 6 targeting site. The sgRNAs were injected into the embryos with Cas9 mRNA at the single-cell stage; **(B)** The targeting site of sgRNA3 was well-conserved from zebrafish to humans; **(C)** Sanger sequencing confirmed the insertion mutation (c.1629_1630inGAATAATACTG; p.Leu544Glufs*1); **(D)** A schematic diagram of the insertion mutation. The smyd4 protein contains four functional domains: two TPR domains (green), one MYND domain (yellow), and one SET domain (purple). The insertion mutation created a truncated mutant protein that lacks the entire C-terminus, which contained the key functional domain TPR2; **(E)** qRT-PCR confirmation that *smyd4* expression is significantly decreased in MZ*smyd4*^L544Efs*1^ embryos; **(F)** Whole-mount *in situ* hybridization analysis confirmed the loss of *smyd4* expression in MZ*smyd4*^L544Efs*1^ embryos at 72 hpf.

To determine the *smyd4* mRNA levels in early to late zebrafish embryos, we performed a qRT-PCR analysis on embryos at the single-cell (0.2 hours post-fertilization (hpf)), 64-cell (2 hpf), blastula (4 hpf), mid-gastrula (8 hpf), late-gastrula (10 hpf), early primordium (24 hpf), long pectoral (48 hpf), and protruding mouth stage (72 hpf) stages (Fig 1A). We found that *smyd4* transcripts were highly present in the single-cell stage, suggesting that a large amount of *smyd4* was present as maternal transcripts. Zygotic *smyd4* transcription peaked at the blastula stage and was followed by downregulation in the gastrula stage, indicating the role of this transcript in zebrafish early development. The overall expression levels of *smyd4* were significantly further downregulated at 48 hpf and followed by a quick reactivation of expression at 72 hpf. We then used whole-mount *in situ* hybridization to determine the spatio-temporal expression pattern of *smyd4* in developing zebrafish embryos. As shown in Fig 1B–1E, *smyd4* was expressed ubiquitously in mid- and late-gastrula embryos; the highest level of expression was found at the polster in late-gastrula embryos (Fig 1D and 1E). By 24 hpf, *smyd4* transcripts were found to be enriched in the developing heart and blood vessel (Fig 1F and 1G) and became more restricted to the cardiovascular system at 48 hpf (Fig 1H–1M). Interestingly, the developing ventricle had a significantly higher level of *smyd4* expression than the atrium (Fig 1J and 1K), suggesting its role in cardiac development. The reactivation of *smyd4* expression at 72 hpf appeared not to be cardiac specific (Fig 2F).

### *smyd4-*deficient zebrafish is generated by using the CRISPR/Cas9 technology

To determine the biological function of *smyd4*, we generated *smyd4-*deficient zebrafish using the CRISPR/Cas9 technology. Three sgRNAs were designed to target different exons of *smyd4* that contribute to the ZMYND, C-terminal TPR2, and SET domains. We were able to generate an 11-nt insertion in exon 6 that was targeted by sgRNA3 (Fig 2A). As shown in Fig 2B, the sgRNA3 targeted sequence is well conserved from zebrafish to humans. Sequencing analysis confirmed this 11-nt insertion, which led to a frameshift at amino acid 545 (*smyd4*^L544Efs*1^) (Fig 2C and 2D and S1 Fig). This frameshift mutation yielded a premature stop codon (TAA), as indicated by the asterisk in Fig 2C, giving rise to a truncated mutant protein that lacks the entire C-terminus containing the TPR2 domain (Fig 2D). qRT-PCR and *in situ* hybridization analyses demonstrated that the *smyd4* mRNA transcript was completely abolished in MZ*smyd4*^L544Efs*1^ embryos (Fig 2E and 2F). The lack of the *smyd4* mRNA transcript was likely due to the well-known effect of nonsense mutation-mediated degradation (NMD) [23], a process common to frameshift mutations. Thus, we concluded that *smyd4*^L544Efs*1^ was a loss-of-function mutation. To better analyze the cardiac defects in MZ*smyd4*^L544Efs*1^ mutants, we crossed the *smyd4*^L544Efs*1^ line with the cardiomyocyte-specific transgenic reporter line Tg(cmcl2:GFP). The entire work presented here was performed using this bi-genic line (*smyd4*^L544Efs*1^;*cmcl2*:*GFP*) with Tg(cmcl2:GFP) as a normal control, unless otherwise indicated in the text.

### The *smyd4* deficiency leads to abnormal cardiac development

Using the heterozygous breeding scheme, we were able to create heterozygous and homozygous *smyd4*^L544Efs*1^ mutant and wild-type embryos. First, we analyzed the survival of *smyd4*^L544Efs*1^ homozygous mutants and found no early lethality in these mutants, as the total amount of surviving *smyd4*^L544Efs*1^ homozygous mutants reached 25% among all three genotypes, which matched the normal Mendelian ratio. These homozygous mutants appeared normal in terms of growth and reproductivity. However, when examining these mutants, we observed a slight increase in embryos with abnormal cardiac situs ambiguus in *smyd4*^L544Efs*1^ homozygous (6 out of 14, 43%) and heterozygous (17 out of 48, 35%) mutants compared to the number of wild-type embryos (2 out of 10, 20%) (S2 Fig). As situs ambiguus is highly relevant for defects in early patterning, this finding prompted us to speculate that the reduced but remaining maternal *smyd4* transcripts in heterozygous females might contribute to the reduced severity of the resulting phenotype. Therefore, we established a homozygous breeding scheme to eliminate the effect of maternal *smyd4* transcripts.

**Fig 3:**
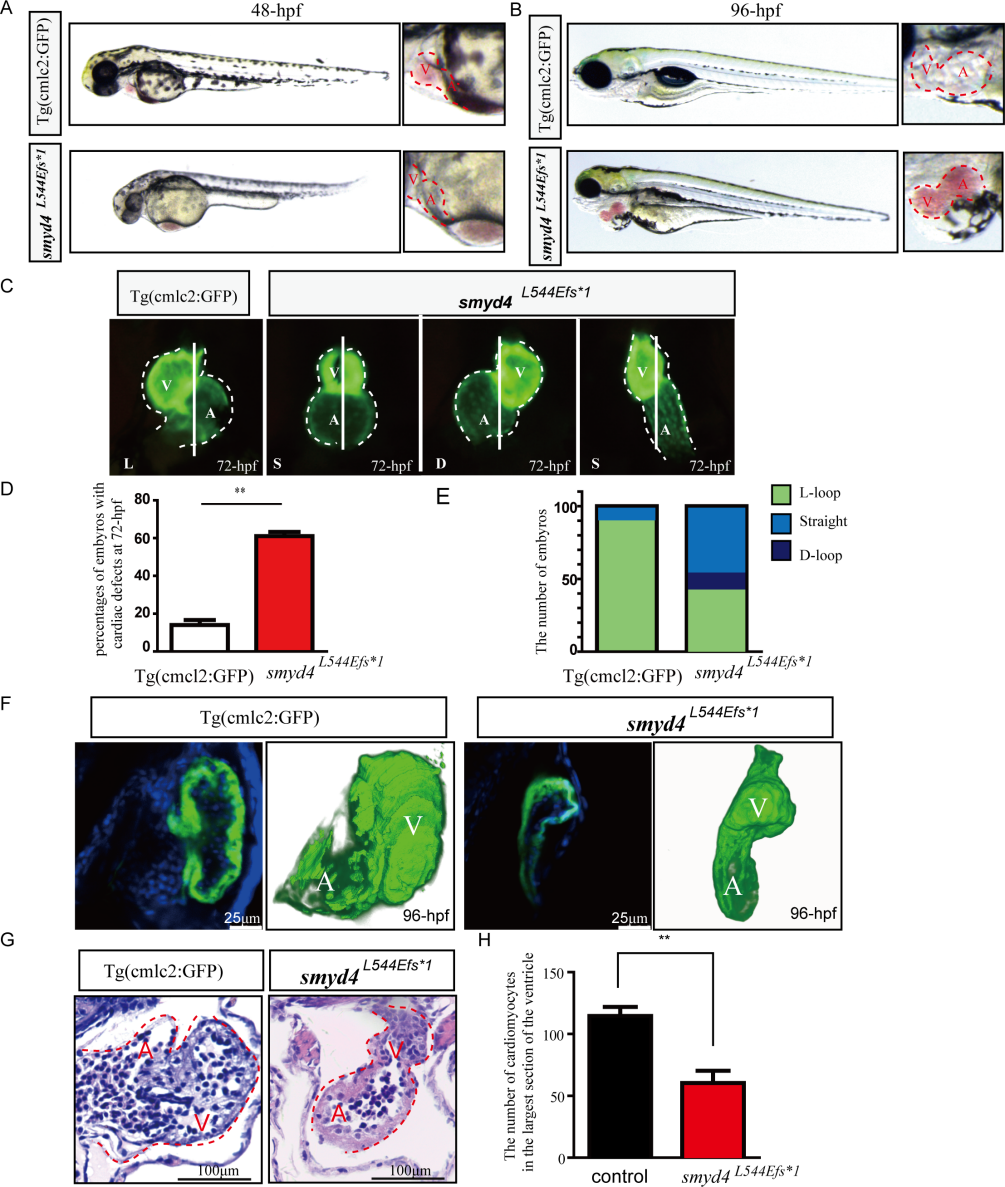
Phenotypical analyses of MZ*smyd4*^L544Efs*1^ embryos. **(A)** Representative images of the MZ*smyd4*^L544Efs*1^ and control embryos at 48 hpf, exhibiting congested blood flow in the ventral vein; **(B)** Representative images of the MZ*smyd4*^L544Efs*1^ and control embryos at 96 hpf, displaying severe pericardial edema; **(C)** Representative images of the MZ*smyd4*^L544Efs*1^ and control hearts at 72 hpf, demonstrating abnormal cardiac morphology and left-right looping defects; **(D)** Significantly more MZ*smyd4*^L544Efs*1^ embryos had abnormal cardiac development than control fish Tg (cmcl2:GFP) (61% ± 4.0% mutants vs. 14% ± 4.6% controls, n = 100, p < 0.01**); **(E)** Comparison of left-right patterning defects in the MZ*smyd4*^L544Efs*1^ (n = 100) and control (n = 100) embryos (green: normal L-loop; blue: straight; dark blue: D-loop). Only 10% of the control embryos showed an abnormal “straight” cardiac left-right asymmetry pattern. However, 48% of MZ*smyd4*^L544Efs*1^ embryos exhibited a “straight” left-right asymmetry pattern, and 11% showed a “D-loop” left-right asymmetry pattern; **(F)** Representative confocal images of the ventricular wall and 3D re-construction images of the MZ*smyd4*^L544Efs*1^ and control hearts at 96 hpf. The confocal scanned images demonstrated hypoplastic wall with significant reduction of trabeculation in MZ*smyd4*^L544Efs*1^ ventricle when compared to the wild-type control ventricle. The 3D-reconstruction of the hearts confirmed left-right looping defects in MZ*smyd4*^L544Efs*1^ hearts (For a better view of 3D-structures, see supplemental material S1 and S2 Movies). **(G)** Histological sections and H&E staining images of the MZ*smyd4*^L544Efs*1^ and control hearts at 96 hpf, demonstrating abnormal ventricular walls and reduced trabeculation; **(H)** The quantitative analysis of the number of cardiomyocytes in the ventricle displayed that MZ*smyd4* ^L544Efs*1^ had significantly reduced cardiomyocytes (p<0.01, **).

**Fig 4:**
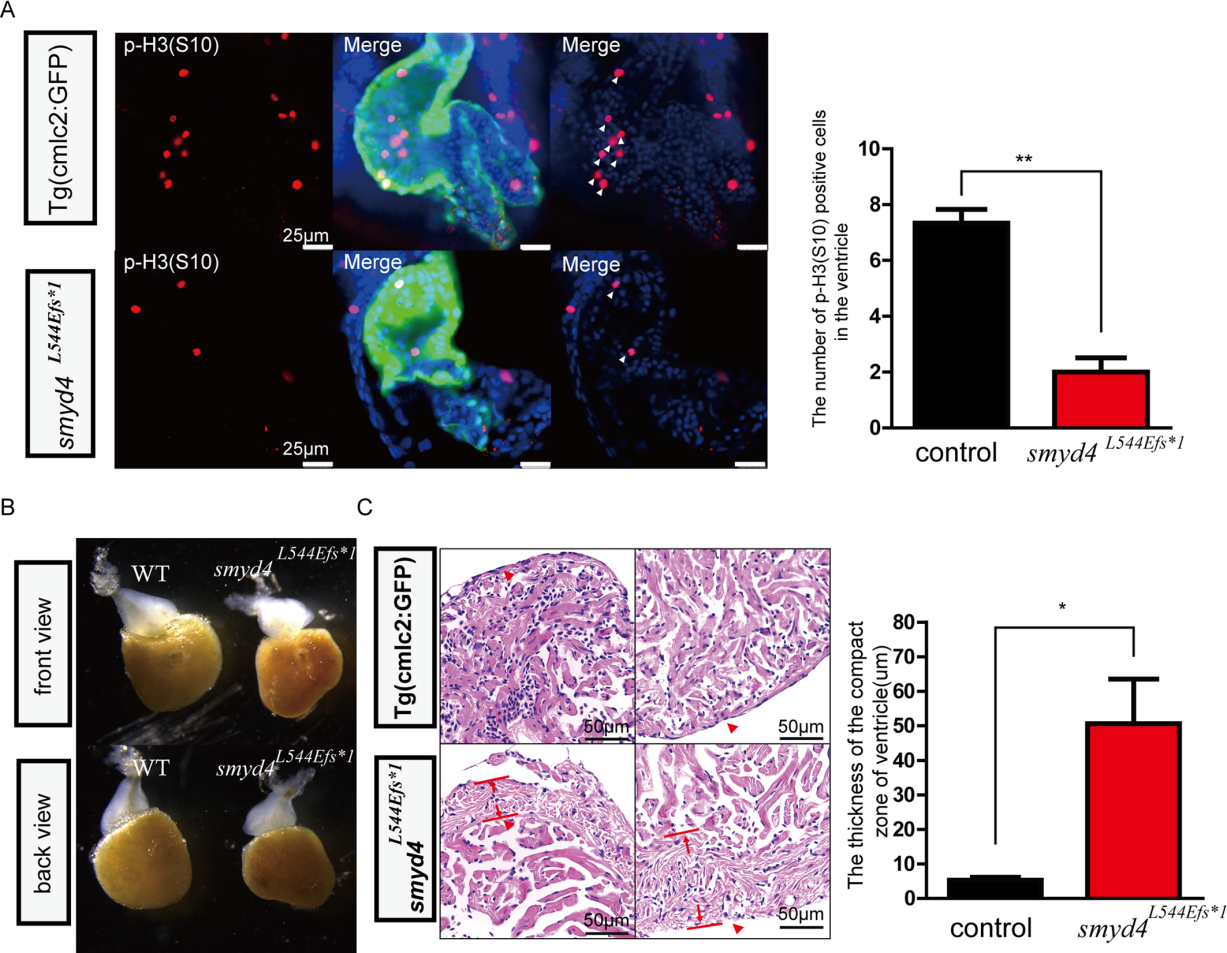
Phenotypical analyses of MZ*smyd4*^L544Efs*1^ embryos. **(A)** The p-H3(S10) immunofluorescence staining showed decreased cell proliferative activity in MZ*smyd4*^L544Efs*1^ cardiomyocytes at 48 hpf. The number of p-H3 (S10) positive cells in the MZ*smyd4*^L544Efs*1^ ventricles was significantly decreased when compared to the control group (p<0.01, **); **(B)** The abnormal ventricular morphology of the adult MZ*smyd4*^L544Efs*1^ hearts compared to the morphology of control adult hearts; **(C)** Histological sections and H&E staining images of the adult MZ*smyd4*^L544Efs*1^ and control hearts, demonstrating significant thickening of the compact ventricular wall. (The red arrows showed that in the similar compact zone of the ventricle). The quantitative analysis displayed that the thickness of the ventricle compact zone was significantly increased in adult MZ*smyd4* ^L544Efs*1^ hearts compared to Tg(cmcl2:GFP) (p<0.05, *).

The MZ*smyd4*^L544Efs*1^ embryos from the homozygous breeding scheme displayed severe pericardial edema (Fig 3A and 3B) and/or congested blood flow in the ventral veins (Fig 3A and 3B), suggesting prominent cardiac defects or dysfunctions associated with the MZ*smyd4*^L544Efs*1^ embryos. The cardiac defects were analyzed carefully. Major defects included a significantly smaller ventricular size and anomalies of cardiac left-right asymmetric patterning or looping in approximately 60% of MZ*smyd4*^L544Efs*1^ embryos at 72 hpf (Fig 3C and 3G), such as situs ambiguus (straight heart tube) and situs inversus (D-loop heart tube) (Fig 3C). The 3D-reconstruction of confocal images of 96 hpf hearts further confirmed this looping defect (Fig 3F and S1 and S2 Movies), as well as hypoplastic ventricles with less trabeculated myocardial structures in the MZ*smyd4*^L544Efs*1^ hearts (Fig 3F and 3G). The number of cardiomyocytes on the largest section plane of the MZ*smyd4*^L544Efs*1^ mutant ventricle was found significantly reduced when compared to the comparable section plane of wild-type control hearts (S3 Fig and Fig 3H), suggesting a great reduction of total number of cardiomyocytes in MZ*smyd4*^L544Efs*1^ hearts. To determine whether reduced cell number was due to the decreased cellular proliferative activity, we used p-H3 and PCNA immune reactivities to respective antibodies as the indicators for the level of cell proliferative activities in mutant developing hearts. As shown in Fig 4A and S4 Fig, MZ*smyd4*^L544Efs*1^ mutant cardiomyocytes had a dramatically reduced level in cellular proliferation when compared to wild-type controls. In addition, we also evaluated the levels of apoptosis in mutant hearts and we found no evidence of increased level of apoptotic cell in MZ*smyd4*^L544Efs*1^ mutant hearts (S5 Fig).

As adults, these MZ*smyd4*^L544Efs*1^ mutants had abnormal gross cardiac morphologies and histologies, typically with less trabecular myocardia and sometimes with a dramatically increased thickness of the ventricular compact wall (Fig 4B and 4C). Despite these cardiac defects, we observed no apparent defects in skeletal muscle development in MZ*smyd4*^L544Efs*1^ mutants examined at 48 and 72 hpf (S6 Fig), suggesting that *smyd4* is lesser important than *smyd1* in vertebrate muscle development [11].

### SMYD4 interacts with HDAC1, and altered histone modification occurs in MZ*smyd4*^L544Efs*1^

**Fig 5:**
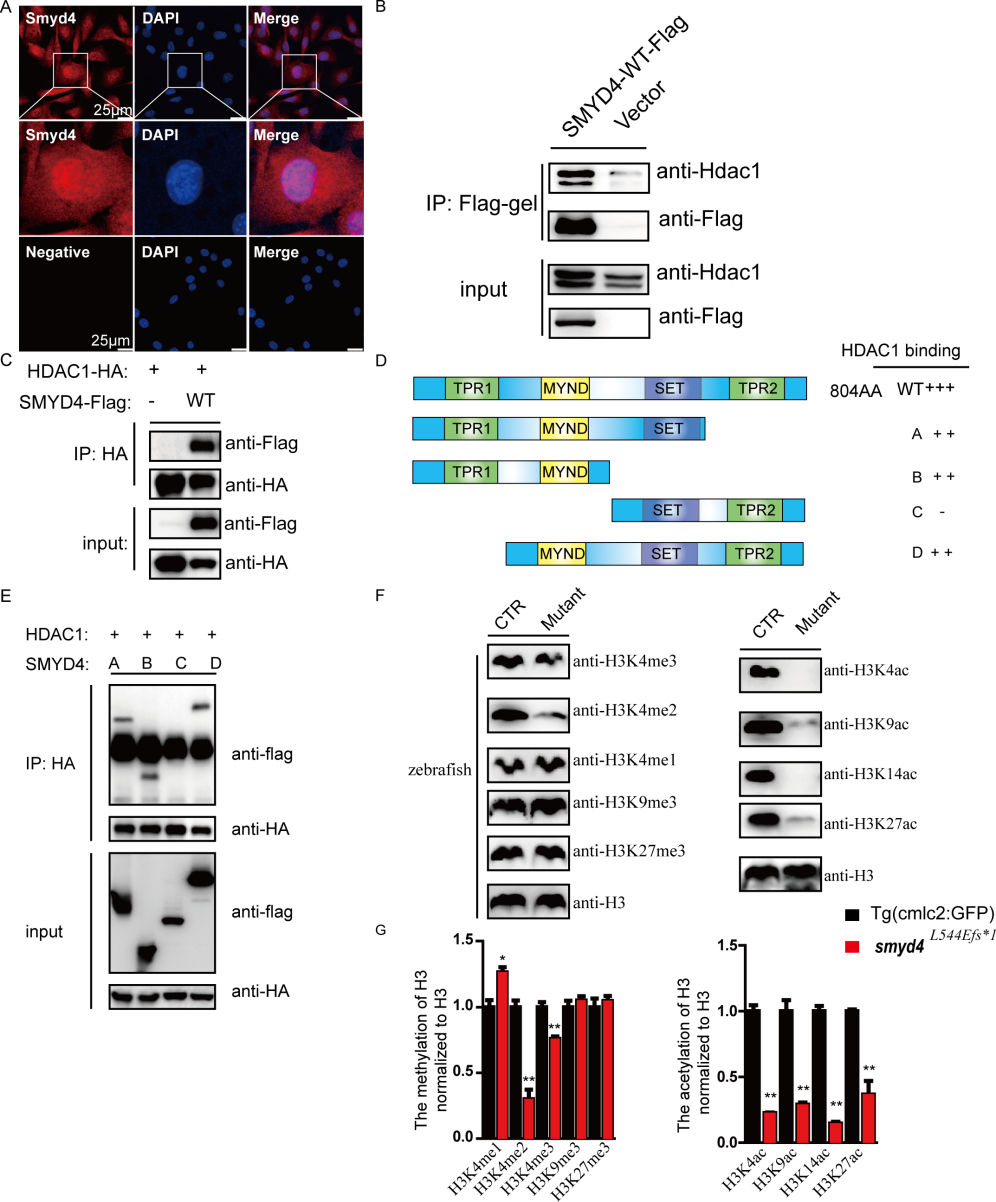
SMYD4 interacts with HDAC1. **(A)** Immunofluorescence staining and representative confocal images of the sub-cellular localization of SMYD4 in HL-1 cells. SMYD4 was found in both the nucleus and cytoplasm. The experimental groups without primary anti-SMYD4 antibody incubation served as negative controls; **(B)** Co-IP/western blotting analysis to confirm the interaction between SMYD4 and HDAC1. SMYD4^flag^ was overexpressed in HL-1 cells. The cell extracts were immunoprecipitated with an anti-FLAG affinity gel, followed by western blotting analysis; **(C)** The interaction between SMYD4 and HDAC1 was further confirmed by Co-IP/western blotting analysis in HEK293T cells; **(D-E)** Using a Co-IP/western blotting assay to map the domains in SMYD4 that are responsible for the interaction with HDAC1. The MYND domain of SMYD4 was found to be critical for this interaction; **(F)** Aberrant histone modifications in MZ*smyd4*^L544Efs*1^ embryos at 48 hpf. Specifically, H3K4me2 and H3K4me3 were significantly reduced, and H3K4me was increased, while H3K9me3 and H3K27me3 were not affected, suggesting that SMYD4 is a H3K4-specific methyltransferase. H3K4ac, H3K9ac, H3K14ac, and H3K27ac were significantly abolished in MZ*smyd4*^L544Efs*1^ mutants, suggesting the increased activity of HDAC1 in MZ*smyd4*^L544Efs*1^ mutants and confirming SMYD4 as an important negative regulator of HDAC1 function; **(G)** The semi-quantitative analysis of the histone modifications changes in MZ*smyd4*^L544Efs*1^ mutants. (p<0.05 *, p<0.01 **).

As shown in Fig 5A, SMYD4 was localized to both the nucleus and cytoplasm. To determine the biochemical function of SMYD4, flag-tagged SMYD4 (SMYD4^flag^) was first overexpressed in the HL-1 mouse cardiomyocyte cell line, followed by immunoprecipitation and mass spectrometric analysis to identify its interacting proteins. HDAC1 was identified as one of the major proteins to interact with SMYD4 (Fig 5B). This finding was further confirmed by Co-IP/western blotting analysis using a HEK293T cell line in which both SMYD4^flag^ and HA-tagged HDAC1 (HDAC1^HA^) were co-overexpressed (Fig 5C). There are four functional domains in SMYD4. Two TPR domains are located at the N- and C- termini, an MYND domain can mediate interactions with partner proteins, and a SET domain functions as a methyltransferase. To determine the functional domain that was responsible for the SMYD4/HDAC1 interaction, as shown in Fig 5D and 5E, we generated several mutations in SMYD4 with different combinations of deletions in the SMYD4 protein and co-expressed these mutants with HDAC1 in HEK293T cells. We were able to use Co-IP/western blotting assays to demonstrate that the MYND domain was responsible for the interaction between SMYD4 and HDAC1.

To confirm the biochemical activities of SMYD4 as a methyltransferase and as a functional partner of HDAC1 in histone modification, we analyzed the changes in both histone methylation and acetylation modifications in the MZ*smyd4*^L544Efs*1^ embryos. As shown in Fig 5F and 5G, di- (me2) and tri-methylation (me3) at the lysine 4 site (K4) of histone 3 (H3) were significantly reduced, and mono-methylation of lysine 4 (H3K4me) was increased, while other lysine residues (*i*.*e*., K9 and K27) were not affected, suggesting that SMYD4 was specifically involved in H3K4 methylation. Interestingly, the acetylation of lysines 4, 9, 14, and 27 was dramatically reduced in the MZ*smyd4*^L544Efs*1^ mutants examined at 48 hpf (Fig 5F and 5G). This finding suggested that SMYD4 is a critical part of the HDAC1 functional complex and may function as an important negative regulator of HDAC1-mediated histone 3 modification and epigenetic regulation. Taken together, these data indicate that SMYD4 is a functional methyltransferase specific for H3K4 methylation and a functional partner of HDAC1 for regulating H3 acetylation.

### Rare *SMYD4* variants associated with CHD patients

**Fig 6:**
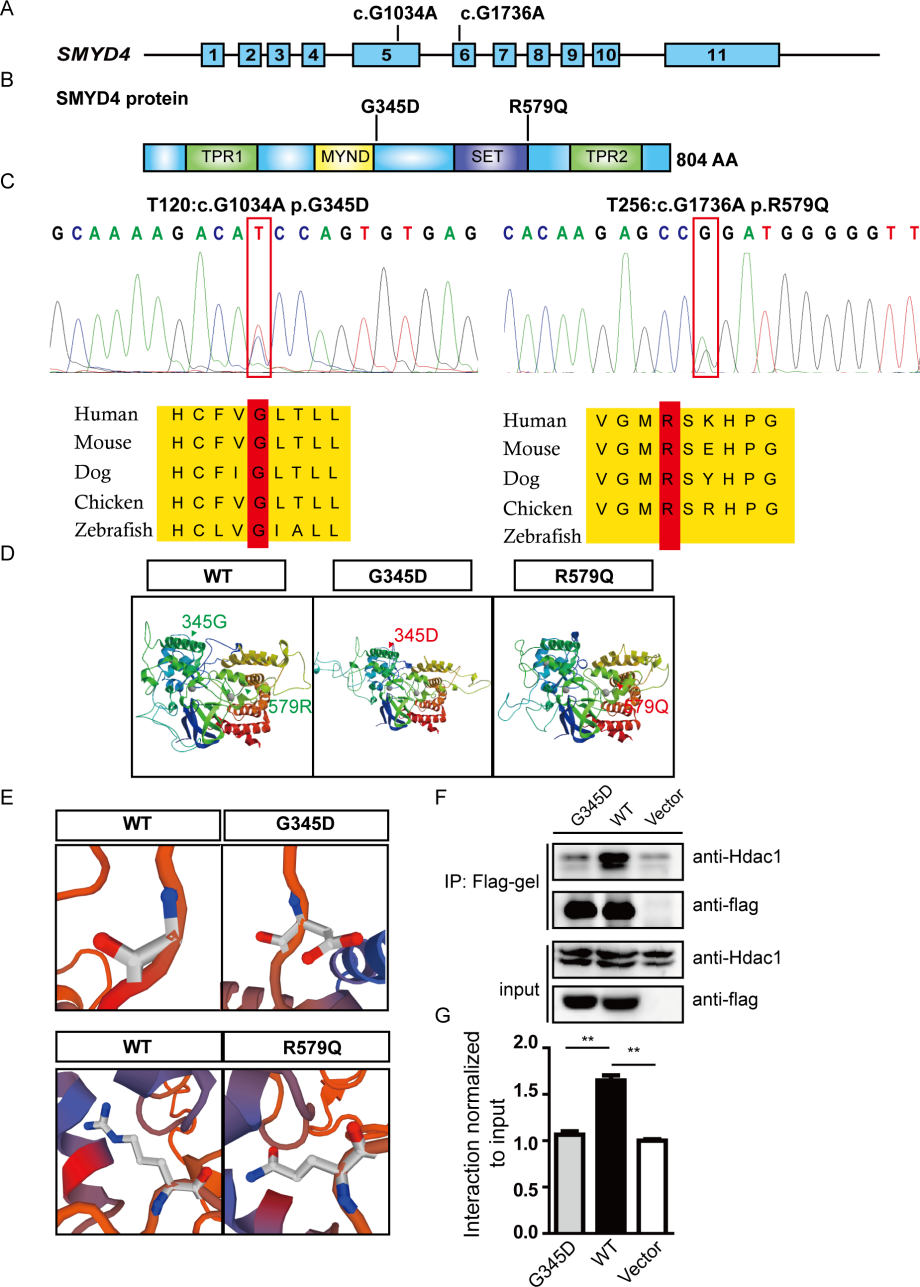
Rare variants in *SMYD4* are associated with CHD patients. **(A)** Schematic diagram of the human *SMYD4* gene. SMYD4 is composed of eleven exons, and two rare missense variants (c. 1034G>A p.G345D and c. 1736G>A p.R579Q) located in exon 5 and 6, respectively, were identified in two CHD patients from a 208-patient cohort; **(B)** The variants G345D and R579Q are located at the edge of two functional domains, the MYND and SET domains, respectively; **(C)** Sanger sequencing confirmed the variants. Both variants were found at a site that is highly conserved from zebrafish to humans; **(D)** Predicted 3D structure of the wild-type and mutant SMYD4 proteins. The structures of both mutant proteins are significantly changed (The arrows pointing the location of the mutant amino acid); (**E**) Comparison of the structures at the mutation sites between wild-type SMYD4 and the two mutants. The structures of the mutant amino acids are completely changed; (**F**) Co-IP/western blotting assays revealed the attenuated interaction between SMYD4(G345D) and HDAC1 in HL-1 cells; (**G**) The semi-quantitative analysis of the Western blotting results demonstrated that the G345D mutant SMYD4 protein significantly disrupted the interaction with HDAC1 compared to the wild-type SMYD4 protein (p<0.01, **).

We used Target Exome Sequencing (TES) and screened a cohort of 208 patients with CHDs for potential genetic variants of *SMYD4*. The patient information is summarized in S1 Table and S2 Table. Two rare missense variants (c.1034G>A, p. G345D and c.1736G>A, p. R579Q) were identified and confirmed by Sanger sequencing in two individual patients (Fig 6A and 6B). The variants were not recorded in the 1000G database or in our internal whole-exome sequencing database generated from more than 3,500 patients without CHDs. The frequencies of G345D and R579Q in the EXAC database are 1/121,404 and 3/120,896, respectively. G345D was identified in a patient who was diagnosed with DCRV/VSD, and R579Q was identified in a patient with TOF (S3 Table). Both sites were evolutionally conserved from zebrafish to humans (Fig 6C). Both variants (G345D and R579Q) were predicted to be highly pathogenic and harmful by SIFT, PolyPhen2 and MutationTaster. Based on 3D-computational structure prediction using the SWISS MODEL, as shown in Fig 6D and 6E, both mutations led to significant changes in protein structure compared with that of the wild-type SMYD4. To confirm the pathogenicity of the variant *SMYD4(G345D)*, Co-IP/western blotting assays were performed, and we found that the biochemical interaction between SMYD4(G345D) and HDAC1 was greatly attenuated compared to the interaction between SMYD4 and HDAC1 in HL-1 cells (Fig 6F and 6G), consistent with the predicted alteration of protein structure and function in SMYD4(G345D).

### Functional confirmation of *SMYD4(G345D)* as a pathogenic mutation

**Fig 7:**
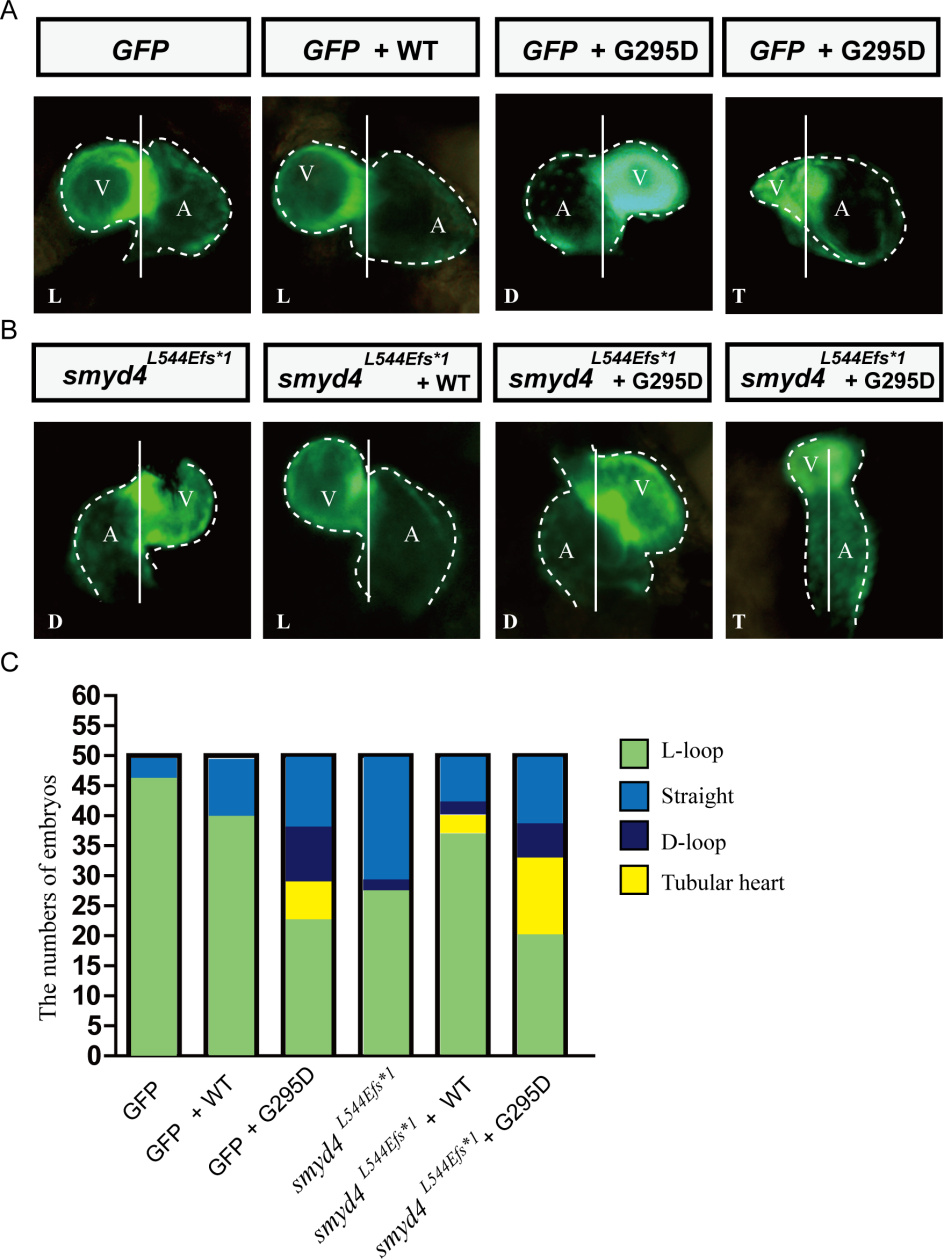
SMYD4(G345D) contributes to cardiac abnormalities. **(A)** Representative cardiac morphology of wild-type embryos injected with either wild-type *smyd4* or *smyd4*(*G295D*) mRNAs; **(B)** Representative cardiac morphology of MZ*smyd4*^L544Efs*1^ embryos injected with either wild-type *smyd4* or *smyd4*(*G295D*); (**C**) Summary of the observations from (A) and (B). A total of 50 fish were screened.

To confirm *SMYD4(G345D*) as a pathogenic mutation, we performed gain-of-function transgenic overexpression experiments by injecting wild-type *smyd4* and *smyd4(G295D)* mRNA into normal Tg(cmcl2:GFP) embryos (Fig 7A and 7C). Based on the amino acid sequences of zebrafish smyd4 and human SMYD4, we generated *smyd4(G295D)* mutant cDNA, which was equivalent to human *SMYD4(G345D)* (Fig 6C and S7 Fig). We found that the *smyd4(G295D)* mRNA caused significantly more embryos with severe heart defects (e.g., D-loop and tubular hearts) than wild-type *smyd4* mRNA (Fig 7A and 7C), suggesting that *SMYD4(G345D)* was harmful to cardiac development.

To further confirm the effects of this mutation, we performed rescue experiments by analyzing and comparing the ability of mutant *SMYD4(G345D)* and wild-type *SMYD4* to rescue the abnormal cardiac phenotypes of MZ*smyd4*^L544Efs*1^ mutants. We injected *smyd4* wild-type and *smyd4(G295D)* mutant mRNAs into MZ*smyd4*^L544Efs*1^ single-cell embryos harvested from the homozygous breeding scheme described above. As shown in Fig 7B and 7C, wild-type *smyd4* mRNA significantly reduced the number of embryos with malformed hearts compared to the number in the MZ*smyd4*^L544Efs*1^ mutant group, which indicates a partial but significant rescue phenotype for the wild-type *smyd4* mRNA. In contrast, the *smyd4(G295D)* mRNA not only failed to rescue MZ*smyd4*^L544Efs*1^ but also significantly increased the number of embryos with malformed hearts and severe cardiac defects, such as tubular hearts (Fig 7B and 7C), further confirming that the *smyd4(G295D)* mutation is pathogenic. Taken together, our data implied that human *SMYD4(G345D)* was deleterious for heart development and a CHD-causing genetic variant.

### Abnormal cardiac gene expression profiling of MZ*smyd4*^L544Efs*1^ hearts

**Fig 8:**
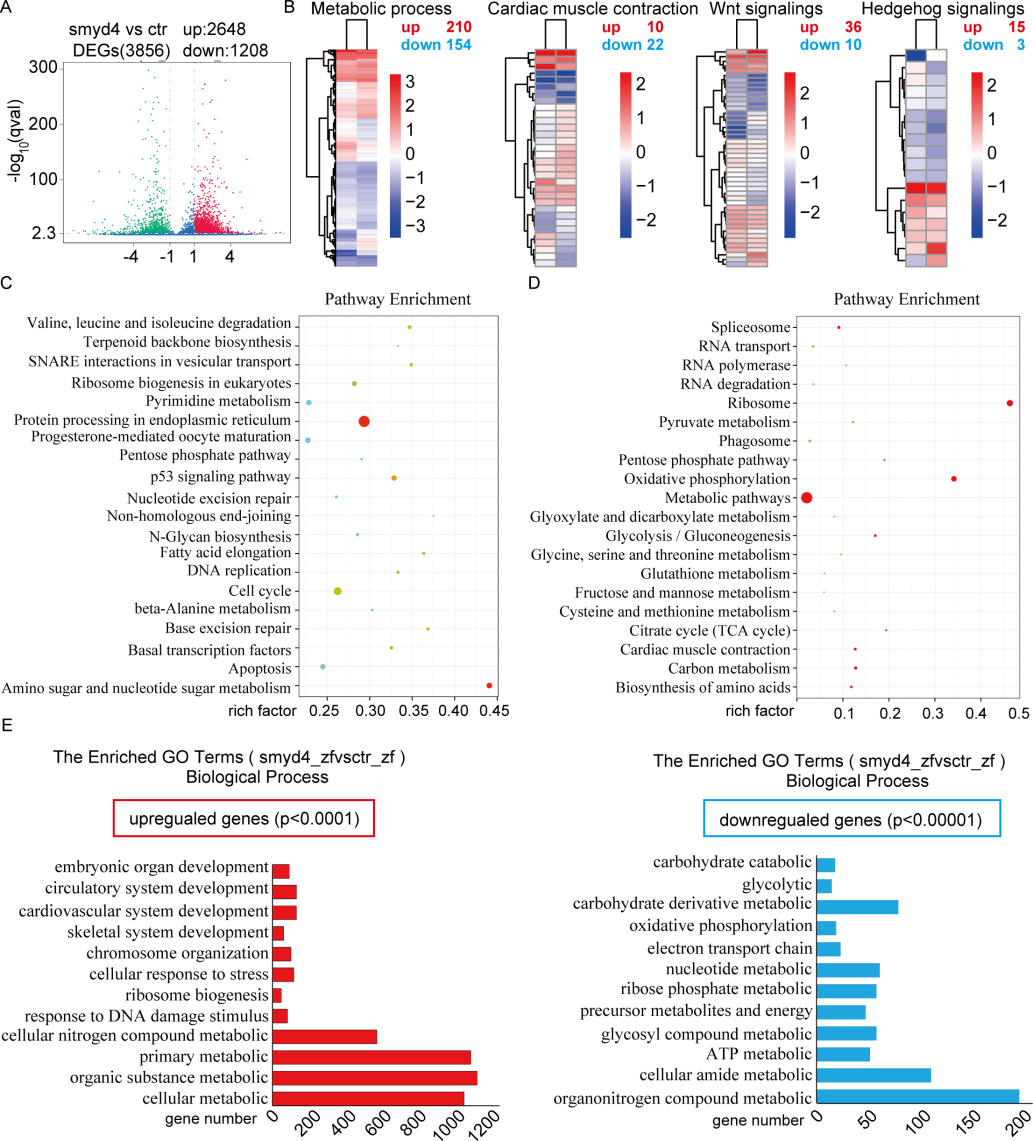
RNA-seq and bioinformatics analysis of MZ*smyd4*^L544Efs*1^ hearts. RNA-seq analyses were performed with Tg(cmcl2:GFP) and MZ*smyd4*^L544Efs*1^ hearts at 72 hpf; **(A)** A volcano plot demonstrated that a total of 3,856 genes were differentially expressed in MZ*smyd4*^L544Efs*1^ hearts (upregulated: 2,648, downregulated: 1,208); **(B)** A heat-map showed that genes involved in cardiac development signaling pathways, including Wnt signaling (upregulated: 36, downregulated: 10), Hedgehog signaling (upregulated: 15, downregulated: 3), the metabolism pathway (upregulated: 210, downregulated: 154), and cardiac muscle contraction (upregulated: 10, downregulated: 22), were dysregulated in MZ*smyd4*^L544Efs*1^ hearts; **(C)** KEGG pathway analysis revealed a major enrichment of upregulated genes in the endoplasmic reticulum-mediated protein processing pathway; **(D)** KEGG pathway analysis revealed an enrichment of downregulated genes in pathways related to metabolic processes and oxidative phosphorylation; **(E)** Gene Ontology (GO) terms of the biological processes (BP) associated with all differentially expressed genes. The up-regulated genes function in organic substance metabolic processes, cardiovascular system development and embryonic organ development (p < 0.0001), while the downregulated genes were enriched in the carbohydrate metabolic process, the glycolytic process, and the ATP metabolic process (p < 0.00001).

Given the severe cardiac defects and abnormal histone modifications in the MZ*smyd4*^L544Efs*1^ embryos, we anticipated a large number of gene alterations in MZ*smyd4*^L544Efs*1^ mutant hearts. We performed RNA-seq analysis, comparing normal and MZ*smyd4*^L544Efs*1^ hearts harvested at 72 hpf. As shown in Fig 8, a total of 3,856 differentially expressed (DE) genes were identified in MZ*smyd4*^L544Efs*1^ hearts. Among those genes, 2,648 genes were upregulated, and 1,208 genes were downregulated (Fig 8A). Not surprisingly, some important genes that were highly relevant to cardiac development were altered, which included the genes involved in cardiac muscle contraction (upregulated: 10; downregulated: 22) and key cardiac signaling pathways, such as the canonical Wnt signaling pathway (upregulated: 36, downregulated: 10) and the Hedgehog signaling pathway (upregulated: 15, downregulated: 3) (Fig 8B). To investigate whether this altered transcriptional profile was associated with specific pathways or biological processes, we performed KEGG pathway analysis, which indicated that the upregulated genes were enriched in the endoplasmic reticulum-mediated protein processing pathway in the Biological Process domain (Fig 8C) and the downregulated genes were enriched in several metabolic pathways, including carbon metabolism and the glycolysis/gluconeogenesis pathway (Fig 8D). Gene ontology (GO) analysis of the upregulated genes also revealed major terms in cellular metabolic processes, in which 975 genes were involved. A GO term analysis of the downregulated genes revealed the enrichment of a large number of genes in the organonitrogen compound metabolic process (185 genes), the ATP metabolic process (49 genes) and the glycosyl compound metabolic process (55 genes) (Fig 8E). This finding provided an important hint that *smyd4*/*hdac1*-mediated epigenetic regulation likely occurred via the control of endoplasmic reticulum-mediated protein processing and several key metabolic pathways in the heart during zebrafish development.

## Discussion

SMYDs are a family of unique lysine-histone methyltransferases that contain the well-conserved SET and MYND domains, as well as two TPR domains. The biological functions of SMYDs are largely unknown. By generating *smyd4* mutant zebrafish using the CRISPR/Cas9 technology, we have shown that *smyd4* is indispensable for cardiac development. This finding is consistent with the observation that ubiquitously expressed *smyd4* in early zebrafish embryos becomes more enriched to zebrafish developing hearts at 48 hpf during embryogenesis, despite the fact that gross expression levels are reduced dramatically at this stage, which is followed by a quick up-regulation of *smyd4* expression in embryos at 72 hpf (Fig 1A). These are critical stages, in which the newly formed heart switches from the cardiac morphogenic pathway to cardiac maturation pathways to eventually form a normal functional heart.

There are several major cardiac defects in the MZ*smyd4*^L544Efs*1^ mutants, including the defects in left-right patterning and looping, and the hypoplastic ventricular walls in the developing hearts. Furthermore, the cell proliferative activities in MZ*smyd4*^L544Efs*1^ developing ventricles are significantly decreased, which likely leads to the hypoplastic ventricle. In adults, the thickening of the ventricular compact wall that is seen in some mutant hearts is likely a maladaptation of compromised cardiac function due to cardiac developmental defects.

Considering *smyd4* shares similar protein structure and enzymatic activities with other SMYD family members, genetic and functional redundancy may occur in MZ*smyd4*^L544Efs*1^ mutants. It has been shown that *smyd1b* mutants display a similar pericardial edema and congestive blood circulation [24]. The morpholino knock-down of another SMYD family member *smyd3* in zebrafish embryos also produces cardiac looping defects [13]. Both of these prior studies suggest potential genetic interactions and/or redundancies among these family members in zebrafish heart development. Some variations in the cardiac defects as well as certain degrees of varying severity of cardiac defects among the mutant embryos further support this notion. However, it is also clear that each member of SMYD family has its unique function. For example, myofibril disorganization in skeletal muscle are only seen in *smyd1b* mutants [24]. We have not observed any such a defect in MZ*smyd4*^L544Efs*1^ mutants, suggesting that the function of *smyd4* is more relevant to cardiac development in zebrafish.

Our observations strongly suggest that *smyd4* plays an important function in cardiac development. This conclusion is also supported by our human genetic study, which identified two rare *SMYD4* variants in a 208-patient cohort with CHDs. The data obtained after the overexpression of mutant *smyd4(G295D)* and from rescue experiments in which *smyd4(G295D)* mRNA was injected into MZ*smyd4*^L544Efs*1^ mutants strongly indicates that the human variant *SMYD4(G345D)* is pathogenic, further suggesting *SMYD4* as a genetic contributor to CHDs. This finding indicates the importance of including *SMYD4* in CHD genetic screening panels in the future.

One of our key findings is that SMYD4 interacts with the major histone modification enzyme and epigenetic regulator HDAC1 via the well-conserved MYND domain. Our finding is consistent with the previous finding that *dSmyd4* can interact with dHDAC1 in *Drosophila* muscle development [15]. The MYND domain is known for its role in protein-protein interactions and was previously shown to recruit the HDAC complex to regulate gene expression [25]. The mutation in *SMYD4(G345D)* is located at the edge of the MYND domain and between the MYND and SET domains. Our biochemical data show that SMYD4(G345D) has a dramatically reduced ability to interact with HDAC1. Although the TPR domains are still poorly understood, previous studies suggest that the C-terminal TPR2 domain is vital for methyltransferase activity and protein-protein interactions [3,26]. The TPR2 domain of SMYD2 is indispensable for its interaction with HSP90, which proved to be critical for titin filament organization [12,27]. Currently, we are in the process of generating *smyd4-tpr2*^*del*^ mutant zebrafish similarly using the CRISPR/Cas9 technology to further investigate the role of TPR2 in *smyd4* biological function.

SMYDs 1 and 3 catalyze mono-, di-, and trimethylation of H3K4 [11,28]. Similarly, SMYD2 can mono-methylate H3K4 (H3K4me) and di-methylate H3K26 (H3K26me2) and p53K37me [6,29]. In MZ*smyd4*^L544Efs*1^ mutants, H3K4me2 and H3K4me3 are reduced, suggesting that *smyd4* is a specific methyltransferase for H3K4 methylation. Notably, H3K4ac, K9ac, K14ac, and K27ac were all abolished in the MZ*smyd4*^L544Efs*1^ mutants. This finding implies that the deficiency of *smyd4* impacts the function of *hdac1* (the gene homologous to both HDAC1 and HDAC2 in mammals). As previously demonstrated, cardiac-specific deletion of the mouse *Hdac1* and *Hdac2* genes evoked a strong heart failure phenotype [20], which is consistent with our finding. We are currently using biochemical approaches to determine whether *smyd4* serves as a simple docking protein to provide chaperone functions for *hdac1* or functions as a key modulating molecule for *hdac1*. Nevertheless, our data suggest that *smyd4* plays a critical role in the epigenetic regulation of gene expression via its dual activities as a methyltransferase and negative regulator of *hdac1*.

RNA-seq analysis comparing wild-type and MZ*smyd4*^L544Efs*1^ mutant hearts demonstrates that the expression of over 3,000 genes is altered, which may reflect the potential function of *smyd4*’s broad epigenetic regulation of its target genes. In addition to genes related to cardiac muscle contraction and cardiac signaling pathways that are highly relevant to cardiogenesis (e.g., the canonical Wnt and Hedgehog signaling pathways) (Fig 8), our KEGG pathway and GO annotation analyses of altered genes revealed an overwhelming enrichment in several cellular metabolic pathways, including the endoplasmic reticulum-mediated protein processing pathway. This finding is very different from the publicly available RNA-seq database for zebrafish heart developmental defects [30–32]. This finding suggests that *smyd4* has a unique and specific biological function in regulating cellular metabolism. However, as it is technically difficult to perform ChIP-seq on zebrafish embryonic hearts, we cannot currently determine which specific components of the pathways are primarily affected and which are affected secondarily. Our future study will switch to a mammalian system to determine the detailed molecular mechanism by which *SMYD4* modulates cellular metabolism or signaling pathways via its important role in epigenetic regulation. We will re-evaluate whether these altered metabolic pathways play critical roles in cardiac development and the cardiac defects seen in *smyd4* mutant embryos.

This study is the first characterization of *SMYD4* in vertebrate development and physiological function. Taken together, our results demonstrate the critical role of *smyd4* in embryonic development and heart formation. Our data suggest that smyd4 functions as a histone methyltransferase and, by interacting with HDAC1, also serves as a potential modulator for histone acetylation. In addition, our work has also provided genetic and functional evidences that rare *SMYD4* variants likely contribute to CHDs.

## Materials and methods

### Ethics statement

All genetic studies were approved by the Ethics Committee of the Children’s Hospital of Fudan University, China. The approval number is: [2015]92). All patients provided written informed consent in accordance to the Declaration of Helsinki. The Research Ethics Committee of the Children’s Hospital of Fudan University, China, approved and monitored all zebrafish procedures following the guidelines and recommendations outlined by the Guide for the Care and Use of Laboratory Animals. The approval number is: [2015]92). For all experiments, wild-type zebrafish embryos of the Tu and transgenic Tg (cmcl2:GFP) (cardiac myosin light chain 2:GFP reporter) strains were used.

### *In situ* hybridization

The expression of *smyd4* was detected in zebrafish embryos from the 10 to 72 hpf stages using a *smyd4*-specific antisense probe. The template for the *smyd4* probe was amplified from the cDNA of zebrafish at 24 hpf. The 508-bp fragment, which was obtained using specific primers (*smyd4*-probe-F: GAAGTGTGTGAAATGTGGAAAGCCTCTT and *smyd4*-probe-R: TTCACTCAGTTCCTGCAGTTCTTCACAG), was cloned into the pEasy-T vector (Promega, USA). After linearization of the plasmids, the antisense and sense probes were transcribed and labelled with digoxigenin *in vitro*. RNA *in situ* hybridization was performed as described previously [33]. Briefly, zebrafish embryos at different stages were collected and fixed in 4% paraformaldehyde at 4°C overnight. Embryos older than 24 hpf were digested by proteinase K at room temperature. Then, the embryos were pro-hybridized at 65°C for 4 hours and subsequently incubated with the antisense or sense probes overnight. An anti-digoxigenin antibody (Roche, USA) was used to bind the probes overnight at 4°C. Finally, the embryos were stained with NBT/BCIP (Vector, USA) and photographed in methylcellulose using a Leica M205C microscope.

### Zebrafish RNA preparation and qRT-PCR

Embryos of the wild-type Tu zebrafish strain were collected at 0.2, 2, 4, 8, 10, 24, 48 and 72 hpf. The total RNA was extracted using the TRIzol reagent (Invitrogen, USA) and converted to cDNA using the PrimeScript RT Reagent Kit (Takara Bio, Japan). The real-time qPCR reactions were performed with SYBR Premix Ex Taq (Takara Bio, Japan) using the Roche 480 plus system (Roche, USA). The real-time primers for the zebrafish are summarized in S4 Table.

### Generation of *smyd4* mutant zebrafish lines

*smyd4* target sites were designed using the website http://zifit.partners.org/ZiFiT/CSquare9Nuclease.aspx. The provided sites were then screened in Ensemble. Three sites that specifically recognize the sequence of *smyd4* in the zebrafish genome were chosen for the interruption of *smyd4*. sgRNA1 (GGAGTAATGAAGCACTGCTG), sgRNA2 (GGAGCTGATCTGCTGGCCAT), and sgRNA3 (GGAGCGTCAGCGCCTCCTGC) targeted exons 2, 4 and 6 of *smyd4*, respectively. We cloned these sites into the gRNA plasmid p-T7-gRNA, which was provided by Professor Li Qiang. gRNAs were transcribed *in vitro* using the MAXIscript T7 kit (Ambion, USA). Cas9 mRNA was transcribed from the pSP6-2Snls-spCas9 plasmid using the SP6 mMESSAGE mMACHINE Kit (Ambion, USA), and poly A tails were added using the poly A Tailing Kit (Ambion, USA). All gRNAs and the Cas9 mRNA were purified and dissolved in nuclease-free water before injection using the mirVana miRNA Isolation Kit (Ambion, USA) and the RNA Purification Kit (TIANGEN, China). gRNA and Cas9 mRNA were co-injected into the embryos at the single-cell stage. Twenty injected embryos were used to identify the efficiency, and the remaining embryos were raised to adulthood to obtain the mosaic founders. These mosaic fish were crossed with wild-type zebrafish to produce heterozygotes, which were genotyped using Sanger sequencing methods. To analyze the cardiac defects in MZ*smyd4*^L544Efs*1^ mutants, we crossed the *smyd4*^L544Efs*1^ line to the cardiomyocyte-specific transgenic reporter line Tg (cmcl2:GFP). All primers and target sites of *smyd4* are summarized in S4 Table.

### Cardiac phenotype analysis of the MZ*smyd4* ^L544Efs*1^ line

Embryos were collected at 48, 72, and 96 hpf for phenotype analysis. The embryos were fixed in methylcellulose and imaged using Leica M205C and Leica SP8 microscopes (Leica, Germany). To determine the number of cardiomyocytes in the developing ventricle, the embryonic hearts at 96 hpf were carefully collected and scanned using Leica SP8 confocal microscope. The z-step was set at 1μm. The images with largest section of ventricles were chosen for the analysis. DAPI and EGFP double positive cells were scored for cardiomyocyte. Adult fish at 6 months of age were photographed to record the body size and the developmental states of different organs, including the head, eyes, fins, and tails. These fish were dissected after anesthesia. The hearts of adult zebrafish were fixed and photographed in 4% paraformaldehyde. Serial sectioning with H&E staining was performed for these heart samples.

### Proliferation and apoptosis assays

Embryos were collected at 48 hpf, fixed in 4% paraformaldehyde at 4°C overnight. For the proliferation assay, the embryos were digested using Collagenase, Type II (Life technologies, USA) at room temperature, and blocking was performed for one hour at room temperature, and followed by incubating with the primary antibody against p-H3(S10) (Abacam, USA) or PCNA (Genetex, USA) at 4°C overnight. Second antibody were from the series of Alexa Fluor (Life technologies, USA). For the apoptosis assay, In Situ Cell Death Detection kit, TMR red (Roche, USA) was used and all procedures were performed as the instruction manual described. Finally, embryo hearts were collected under a Leica M205C stereomicroscope and were imaged using a Leica SP8 microscope.

### Plasmid construction and protein-protein interaction identification

A plasmid containing wild-type human *SMYD4*(BC035077) was obtained from Abmgood (Abmgood, USA). Then, Flag-tagged wild-type *SMYD4* and HA-tagged wild-type HDAC1 were cloned into the expression plasmid. The mutation (G345D) identified in patients was obtained using the KOD-Plus Mutagenesis Kit (Toyobo, Japan). All plasmids were confirmed via Sanger sequencing. The anti-SMYD4 (Proteintech, USA), anti-HDAC1 (Proteintech, USA), anti-Flag (Abmart, China), and anti-HA (Abmart, China) antibodies were used. Wild-type SMYD4 was overexpressed in HL-1 cells. After 48-h transfections, cell lysates were obtained in RIPA containing 1 mM PMSF and complete protease inhibitors (Roche, USA). Immunoprecipitation was performed using an anti-Flag affinity gel (Biotool, USA). SDS-PAGE was performed to resolve the eluates. After sliver staining, the proteins underwent mass spectrometry analysis. Protein-protein interactions were verified in HL-1 cells after transient overexpression of SMYD4. Co-immunoprecipitation was performed to confirm the interaction between SMYD4 and HDAC1 in HEK293T cells using anti-tag antibodies.

### Domain mapping and mutant protein analysis

HEK293T cells were transiently co-transfected with pCDH-Flag-SMYD4 deletion mutants and pcDNA3-HA-HDAC1. Plasmids of *SMYD4* were co-transfected into HEK293T cells, and cell extracts were prepared as described above. Immunoprecipitations were performed with anti-HA affinity beads (Biotool, USA). The beads were washed five times, and bound proteins were eluted in SDS-PAGE loading buffer and analyzed via western blotting.

### Immunofluorescence

Briefly, the immunofluorescence process is described as follows. The cells were fixed in 4% paraformaldehyde and then underwent cell permeation and blocking. The antibody used for immunofluorescence was anti-SMYD4 (Proteintech, USA). Primary antibodies were incubated overnight at 4°C. Secondary antibodies were from the Alexa Fluorescence Series (Life technologies, USA). Finally, the cells were imaged in Diamond anti-fade agent (Life technologies, USA) using a Leica SP8 microscope.

### Zebrafish western blotting and antibodies

Tg(cmcl2:GFP) and MZ*smyd4*^L544Efs*1^ embryos were collected at 48 hpf. Protein was obtained in RIPA containing 1 mM PMSF and complete protease inhibitors (Roche, USA) after sonication. The histone modification antibodies used in this study include anti-H3 (CST, USA), anti-H3K4ac (Active Motif, USA), anti-H3K9ac (Active Motif, USA), anti-H3K14ac (Active Motif, USA), anti-H3K27ac (Active Motif, USA), anti-H3K4me1 (Active Motif, USA), anti-H3K4me2 (Active Motif, USA), anti-H3K4me3 (Abcam, USA), anti-H3K9me3 (Abcam, USA), and anti-H3K27me3 (Millipore, USA).

### Human samples and ethics statement

This study was approved by the research ethics committee of the Children’s Hospital of Fudan University in Shanghai, China (number: [2015]92). The diagnosis of CHD patients was based on echocardiography at the Children’s Hospital of Fudan University in Shanghai, China. All patients involved in this research had not been diagnosed with extra cardiac anomalies and did not have common chromosomal anomalies, such as the 22q11 microdeletion. Human cardiac tissue samples from CHD patients were obtained from the Biobank of the Children’s Hospital of Fudan University in Shanghai, China. Cardiac tissues were removed from the blocked right ventricular outflow tract during surgery. All tissue samples were maintained in RNAlater RNA Stabilization Solution (Thermo Scientific, USA) after surgery and stored at -80°C before use. RNA was extracted from the tissue samples using the Trizol reagent (Invitrogen, USA) and immediately underwent reverse transcription using the PrimeScript RT Reagent Kit (Takara, Japan). All detailed patient information is summarized in S2 Table.

### Human genomic sequencing studies and *in silico* analysis

The peripheral venous blood samples from 113 patients were prepared for DNA extraction using the Blood Extraction Kit (QIAGEN, Germany). The cardiac tissue samples from 95 patients were prepared for cDNA extraction. All of the regions covered by TES and all primers for smyd4 exon sequencing in cDNA are listed in S5 Table. Variant analysis was performed using the Mutation Surveyor software (Softgenetics, USA). All variants were screened in public databases, including the 1000 Genome database, the dbSNP database, and the ExAC database, and an internal database in the molecular diagnosis laboratory at the Children’s Hospital of Fudan University. A risk analysis of SNVs was performed using SIFT, Polyphen2, and Mutation Taster to predict the possible effects on protein function. A 3D structure analysis of the wild-type and mutant proteins was performed on the SWISS MODEL website (https://www.swissmodel.expasy.org/).

### Microinjection of wild-type or mutant *smyd4* mRNA (G295D) and phenotype analysis

Based on its pathogenic prediction, the G345D *SMYD4* variant was selected for mutation analysis. Homology analysis showed that the mutant G295D in *smyd4* was equivalent to the mutant G345D in *SMYD4*. The template for *smyd4* wild-type mRNA transcription was amplified from zebrafish cDNA at 24 hpf. The template of the mutant (G295D) in *smyd4* transcription was obtained using the KOD-Plus Mutagenesis Kit (Toyobo, Japan). The wild-type and mutant mRNAs of *smyd4* were transcribed using the mMessage mMachine T7 Ultra Kit (Ambion, USA), and poly A tails were added using the poly A Tailing Kit (Ambion, USA), according to the instruction manual. The mRNAs were resolved in nuclease-free water and finally quantified to 150 ng/μl for microinjection. Embryos of Tg(cmcl2:GFP) and MZ*smyd4* ^L544Efs*1^ were collected. A total of 3 nl of mRNA was microinjected into embryos at the single-cell stage. Fifty embryos from each group with cardiac GFP at 48 hpf were chosen for phenotype analysis. The embryos were fixed in 3% methylcellulose after anesthetization and then observed for cardiac morphology using a Leica M205C stereomicroscope.

### RNA sequencing

Heart tissue was collected from 50 MZ*smyd4*^L544Efs*1^ or Tg(cmcl2:GFP) embryos. Cardiac-specific GFP helped us successfully obtain embryo heart tissue. Embryo hearts were collected at 72 hpf after anesthesia using a Leica M205C stereomicroscope. In-depth RNA sequencing was performed by the Novogene Experimental Department in China. The raw sequencing image data were examined via the Illumina analysis pipeline and aligned with the unmasked zebrafish reference genome. Differential expression analysis of the two groups was performed using the DESeq2 R package (1.10.1). The resulting P-values were adjusted using the Benjamini and Hochberg’s approach for controlling the false discovery rate. Genes with an adjusted P-value < 0.005 and |log_2_ (Fold change)|>1 found by DESeq2 were assigned as differentially expressed.

### Statistical analyses

The Student’s t-test was used for all statistical analyses. A p-value of < 0.05 (2-sided) was regarded as statistically significant. All experiments were repeated three times. All data were analyzed with GraphPad Prism (version 5.0).

## Supporting information

S1 Fig Genotyping of MZ*smyd4*^L544Efs*1^ mutants. An 11-nt insertion was achieved using the CRISPR-Cas9 technology and was validated by cDNA PCR and sequencing analyses in MZ*smyd4*^L544Efs*1^.
(TIF)

S2 Fig Heterozygous breeding scheme generated some abnormal cardiac situs ambiguus and hypoplastic hearts in homozygous and heterozygous mutants. (TIF)

S3 Fig Representative images for analyzing the number of cardiomyocytes in MZ*smyd4*^L544Efs*1^ mutant and control hearts. The confocal images of the largest section of the ventricle of MZ*smyd4* ^L544Efs*1^ and Tg(cmcl2:GFP) hearts at 96 hpf.
(TIF)

S4 Fig The PCNA immunofluorescence staining showed decreased cell proliferative activity in MZ*smyd4*^L544Efs*1^ cardiomyocytes at 48 hpf. (TIF)

S5 Fig Cellular apoptosis was not seen in the MZ*smyd4*^L544Efs*1^ ventricles at 48 hpf. TUNEL assays showed that there is no apoptosis in neither control hearts and MZ*smyd4*^L544Efs*1^ hearts (Upper panel). The positive TUNEL signals (red fluorescence) in the tail fin and embryos at 48 hpf treated with DNase I were used as positive controls for the analysis (Lower panel).
(TIF)

S6 Fig Confocal images of fluorescence immunostaining of actin1a, co-stained with DAPI. The skeletal muscle structures of MZ*smyd4*^L544Efs*1^ mutants appeared to be normal at 72 hpf.
(TIF)

S7 Fig Schematic domain alignment and homology between the human and zebrafish SMYD4 proteins. Both human SMYD4 and the zebrafish smyd4 protein have four functional domains, including 2 TPR domains, one MYND domain, and one SET domain. These domains are highly conserved between the two species. The two rare variants identified in CHD patients are located at the edge of the MYND and SET domains. Zebrafish smyd4(G295D) is equivalent to human SMYD4(G345D).
(TIF)

S1 Table Clinical characteristics of CHD patients. (DOCX)

S2 Table Detailed CHD patient information. (XLSX)

S3 Table Information regarding the rare variants identified in the CHD patients. (DOCX)

S4 Table Primers used for qPCR, CRISPR/Cas9 construction, and genotyping in zebrafish. (XLSX)

S5 Table All of the regions covered by TES and sequencing primers for Sanger sequencing. (XLSX)

S1 Movie 3D reconstruction of hearts in Tg(cmcl2:GFP) embryos. (MP4)

S2 Movie 3D reconstruction of hearts in MZ*smyd4* ^L544Efs*1^. (MP4)
