# Supplementary figures and images for: The roles of SMYD4 in epigenetic regulation of cardiac development in zebrafish
Source: PLoS Genet. 2018 Aug 15;14(8):e1007578. doi: 10.1371/journal.pgen.1007578 (PMC6110521; doi:10.1371/journal.pgen.1007578)

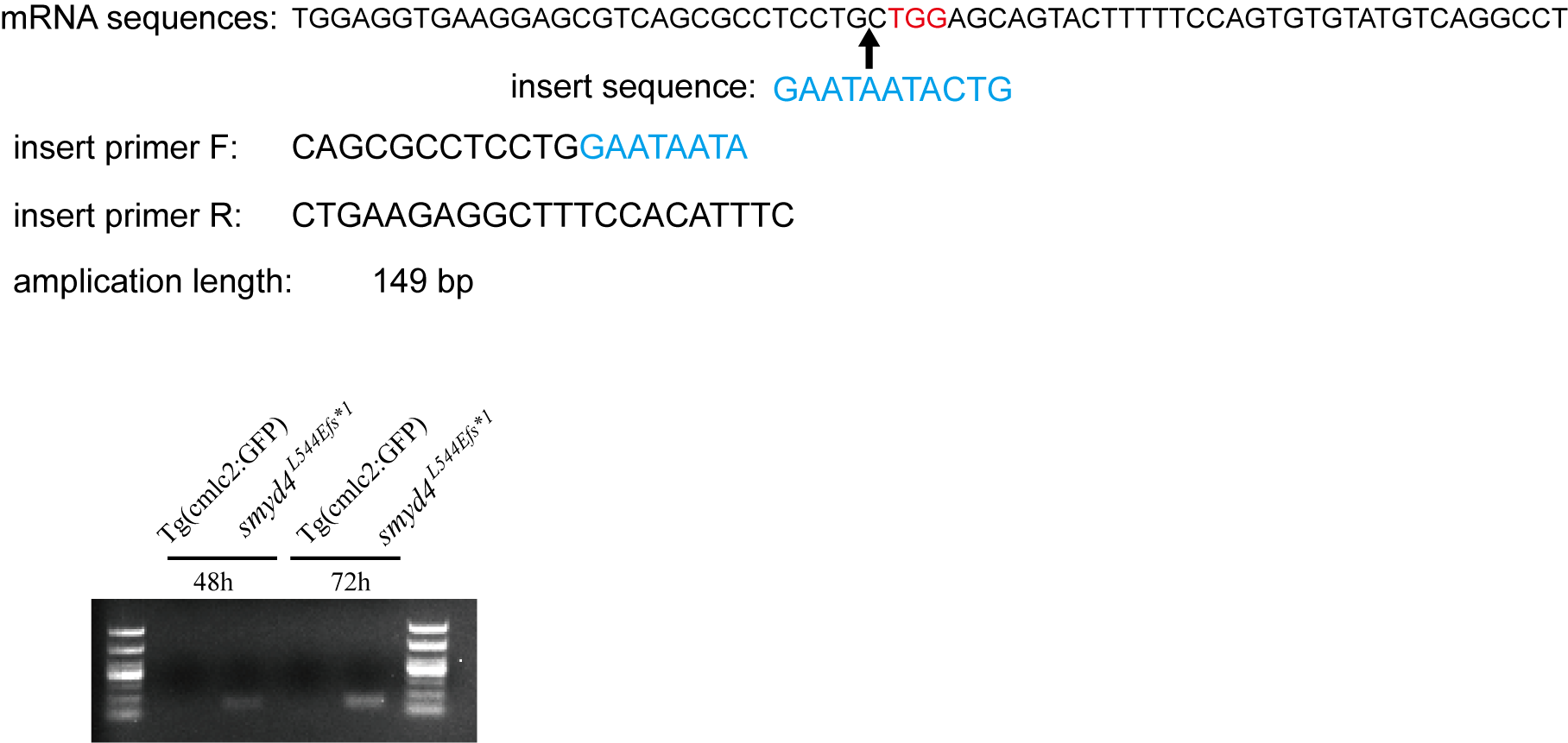

Supplement: S1 Fig — An 11-nt insertion was achieved using the CRISPR-Cas9 technology and was validated by cDNA PCR and sequencing analyses in MZsmyd4L544Efs*1. (TIF) [file pgen.1007578.s001.tif]

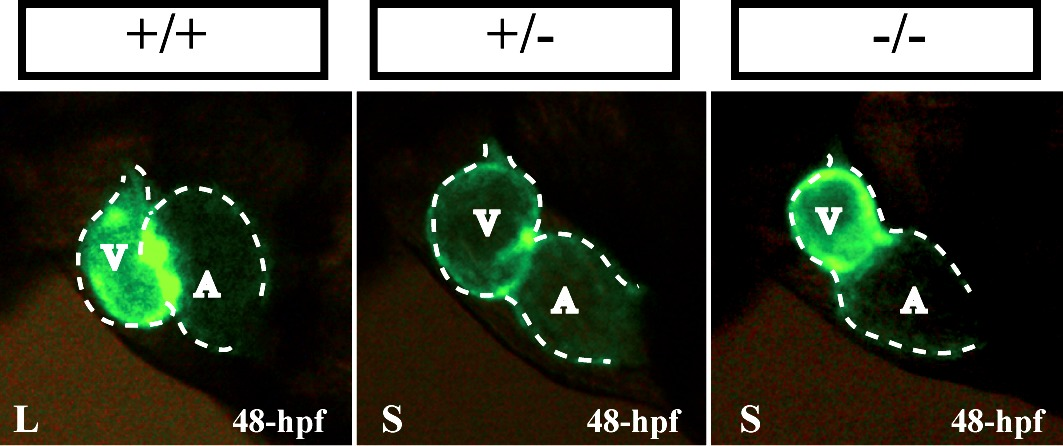

Supplement: S2 Fig — (TIF) [file pgen.1007578.s002.tif]

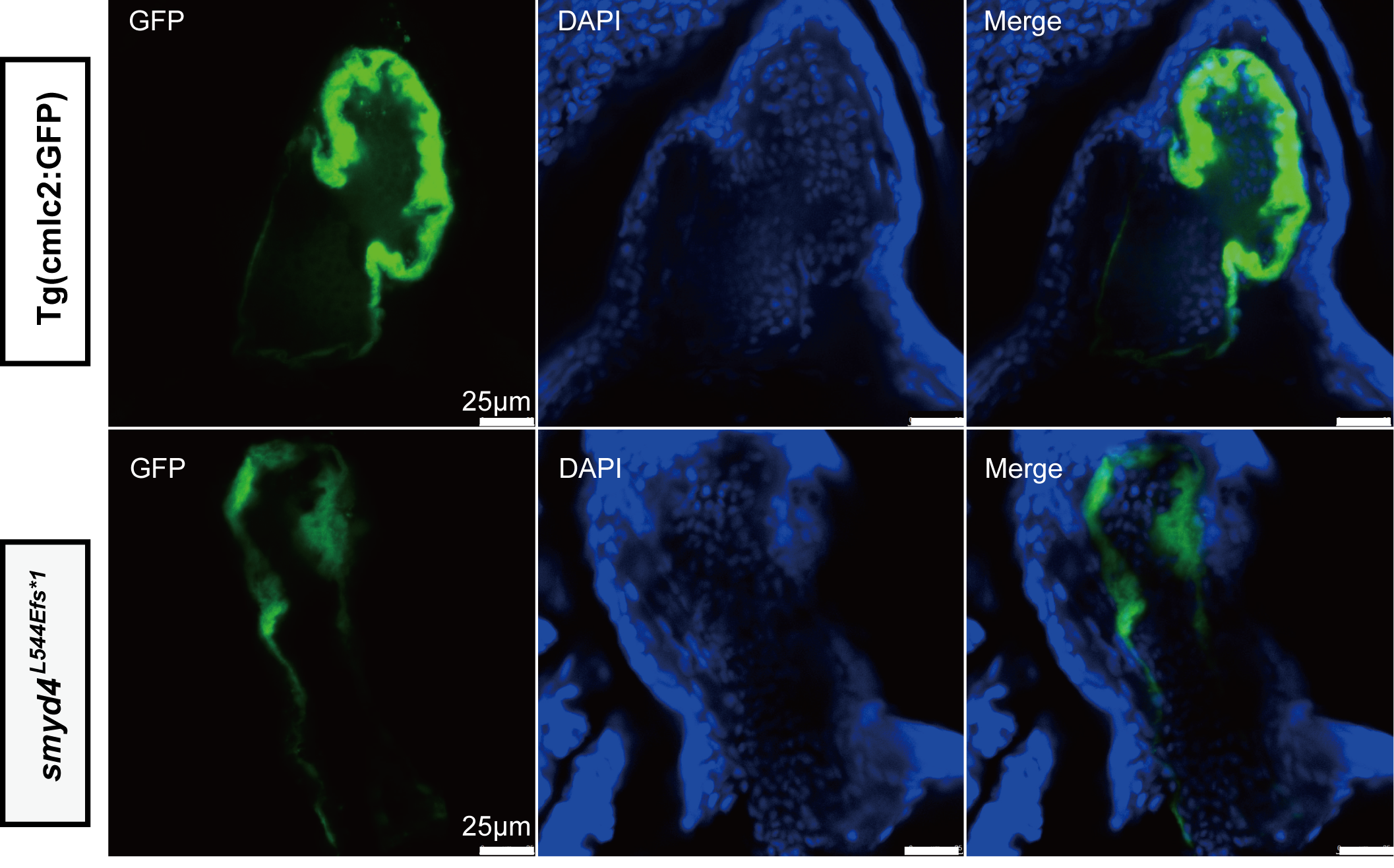

Supplement: S3 Fig — The confocal images of the largest section of the ventricle of MZsmyd4 L544Efs*1 and Tg(cmcl2:GFP) hearts at 96 hpf. (TIF) [file pgen.1007578.s003.tif]

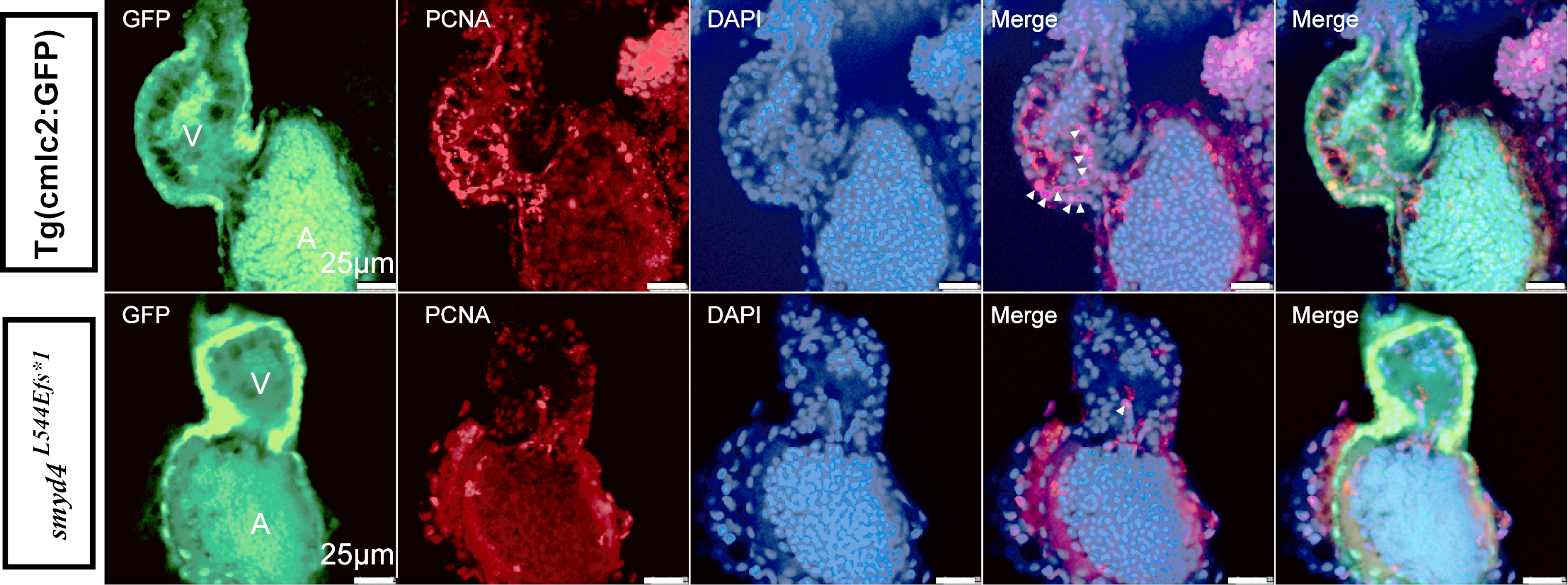

Supplement: S4 Fig — (TIF) [file pgen.1007578.s004.tif]

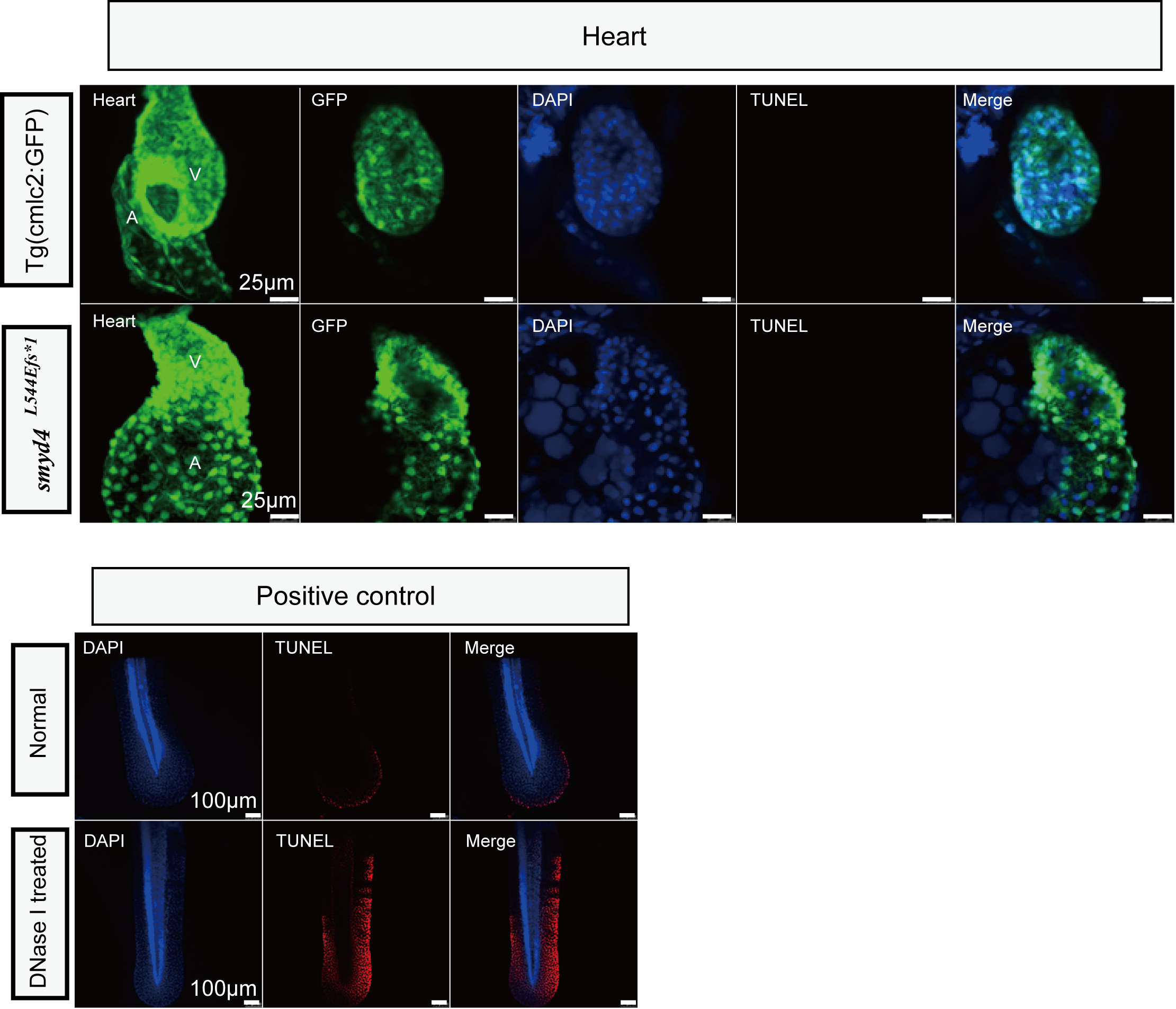

Supplement: S5 Fig — TUNEL assays showed that there is no apoptosis in neither control hearts and MZsmyd4L544Efs*1 hearts (Upper panel). The positive TUNEL signals (red fluorescence) in the tail fin and embryos at 48 hpf treated with DNase I were used as positive controls for the analysis (Lower panel). (TIF) [file pgen.1007578.s005.tif]

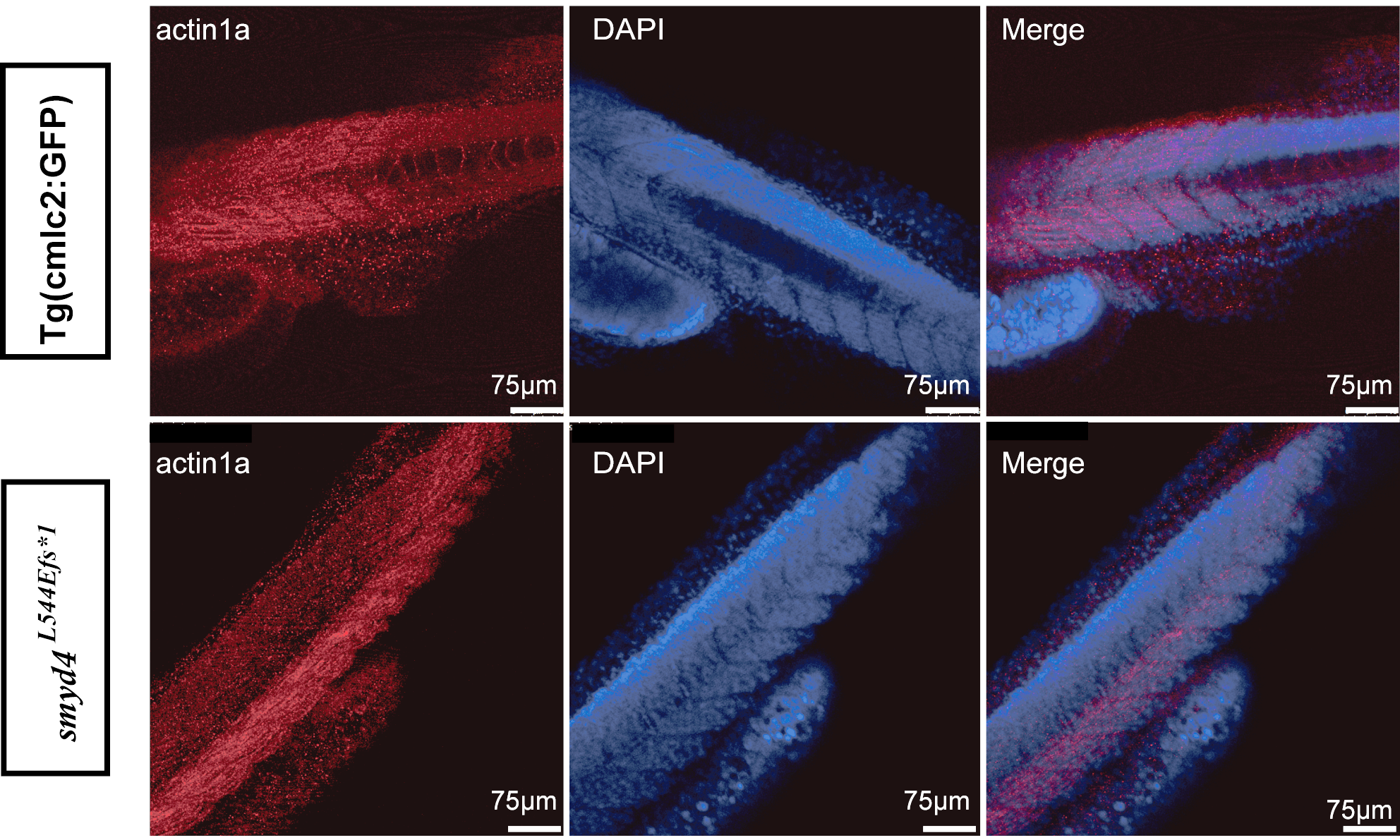

Supplement: S6 Fig — The skeletal muscle structures of MZsmyd4L544Efs*1 mutants appeared to be normal at 72 hpf. (TIF) [file pgen.1007578.s006.tif]

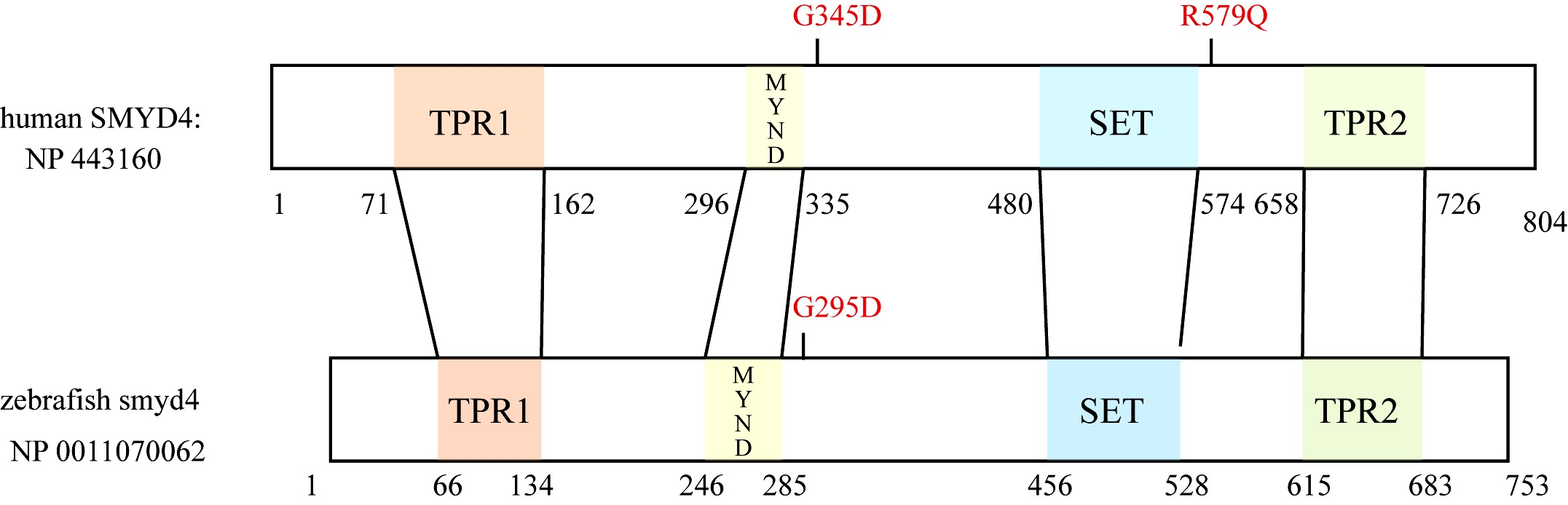

Supplement: S7 Fig — Both human SMYD4 and the zebrafish smyd4 protein have four functional domains, including 2 TPR domains, one MYND domain, and one SET domain. These domains are highly conserved between the two species. The two rare variants identified in CHD patients are located at the edge of the MYND and SET domains. Zebrafish smyd4(G295D) is equivalent to human SMYD4(G345D). (TIF) [file pgen.1007578.s007.tif]
