# Supplementary material for: The roles of SMYD4 in epigenetic regulation of cardiac development in zebrafish
Source: PLoS Genet. 2018 Aug 15;14(8):e1007578. doi: 10.1371/journal.pgen.1007578 (PMC6110521; doi:10.1371/journal.pgen.1007578)
Supplement: S1 Table — (DOCX) [file pgen.1007578.s008.docx]

**S1 Table: Clinical characteristics of CHD patients.**

| Subgroup | Number | Percentage/ Range |
| --- | --- | --- |
| Male  Female | 130  78 | 62.5%  37.5% |
| Age at diagnosis (months) | 26.6 | 1-156 |
| Diagnosis types of TOF  TOF  ASD  VSD  DORV  DCRV | 107  30  24  35  12 | 51.4%  14.4%  11.6%  16.8%  5.8% |

Tetralogy of Fallot, TOF; Atrial septum defect, ASD; Ventricular septum defect, VSD; Double-outlet right ventricle, DORV; Patent ductus arteriosus, PDA; Double-chambered right ventricle, DCRV.
