# Supplementary material for: The roles of SMYD4 in epigenetic regulation of cardiac development in zebrafish
Source: PLoS Genet. 2018 Aug 15;14(8):e1007578. doi: 10.1371/journal.pgen.1007578 (PMC6110521; doi:10.1371/journal.pgen.1007578)
Supplement: S3 Table — (DOCX) [file pgen.1007578.s010.docx]

**S3 Table. Information regarding the rare variants identified in the CHD patients.**

| Gene | Nucleotide change | Amino acid change | | Patient ID | Diagnosis | SIFT/PolyPhen/  MutationTaster | ExAC/1KG  (frequency) | CHD  Patients |
| --- | --- | --- | --- | --- | --- | --- | --- | --- |
| *SMYD4* | c.G1034A | | G345D | T120 | DCRV+VSD | D/D/D | 0.000008/0 | 1/208 |
|  | c.G1736A | | R579Q | T256 | TOF | T/D/D | 0.00002/0 | 1/208 |

Tetralogy of Fallot, TOF; Ventricular septum defect, VSD; Double-chambered right ventricle, DCRV. SIFT, “D” meaning deleterious, score less than 0.05, “T” meaning tolerated, score greater than or equal to 0.05; PolyPhen2, “D” meaning probably damaging, 0.957≤score≤1, “P” meaning likely damaging, 0.453≤score≤0.956, “B” meaning benign, 0≤score≤0.452; MutationTaster, “D” represents as disease causing meaning likely deleterious, “P” represents polymorphism automatic meaning known harmless. ExAC, Exome Aggregation Consortium; 1KG, 1000 genome; “0” in frequency means didn’t find in the database.
